# Transcriptomic diversity of amygdalar subdivisions across humans and nonhuman primates

**DOI:** 10.1126/sciadv.adw1029

**Published:** 2025-09-17

**Authors:** Michael S. Totty, Rita Cervera Juanes, Svitlana V. Bach, Lamya Ben Ameur, Madeline R. Valentine, Evan Simons, McKenna D. Romac, Hoa Trinh, Krystal Henderson, Ishbel Del Rosario, Madhavi Tippani, Ryan A. Miller, Joel E. Kleinman, Stephanie Cerceo Page, Arpiar Saunders, Thomas M. Hyde, Keri Martinowich, Stephanie C. Hicks, Vincent D. Costa

**Affiliations:** ^1^ Department of Biostatistics, Johns Hopkins Bloomberg School of Public Health, Baltimore, MD, USA.; ^2^ Department of Translational Neuroscience, Center for Precision Medicine, Wake Forest University School of Medicine, Winston-Salem, NC, USA.; ^3^ Lieber Institute for Brain Development, Johns Hopkins Medical Campus, Baltimore, MD, USA.; ^4^ Vollum Institute, Oregon Health and Science University, Portland, OR, USA.; ^5^ Division of Neuroscience, Oregon National Primate Research Center, Beaverton, OR, USA.; ^6^ Division of Developmental and Cognitive Neuroscience, Emory National Primate Research Center, Atlanta, GA, USA.; ^7^ Department of Neurology, Johns Hopkins School of Medicine, Baltimore, MD, USA.; ^8^ Department of Psychiatry and Behavioral Sciences, Johns Hopkins School of Medicine, Baltimore, MD, USA.; ^9^ The Solomon H. Snyder Department of Neuroscience, Johns Hopkins School of Medicine, Baltimore, MD, USA.; ^10^ Johns Hopkins Kavli Neuroscience Discovery Institute, Baltimore, MD, USA.; ^11^ Department of Biomedical Engineering, Johns Hopkins University, Baltimore, MD, USA.; ^12^ Center for Computational Biology, Johns Hopkins University, Baltimore, MD, USA.; ^13^ Malone Center for Engineering in Healthcare, Johns Hopkins University, Baltimore, MD, USA.; ^14^ Department of Psychiatry and Behavioral Sciences, Emory University, Atlanta, GA, USA.

## Abstract

The amygdaloid complex mediates learning, memory, and emotions. Understanding cellular and anatomical features specialized in the primate amygdala versus other mammals requires a systematic, anatomically resolved molecular analysis of neuron types. We analyzed five nuclear subdivisions of the primate amygdala with single-nucleus RNA sequencing in macaques, baboons, and humans to examine gene expression profiles for excitatory and inhibitory neurons. Integrated analyses across species identified diverse subtypes of glutamatergic and GABAergic neurons that are highly conserved across primates. Compositional analyses revealed that subdivisions of the primate basolateral complex contain distinct classes of glutamatergic neurons and divergent gene expression profiles for parvalbumin and somatostatin GABAergic neurons. Referencing primate neuron types to transcriptomic atlases of the murine amygdala highlighted primate-specific specializations for glutamatergic neurons and identified which neuron types are conserved across mammals and amygdalar subdivisions. Understanding the molecular heterogeneity of anatomically resolved amygdalar neuron types provides a cellular framework for improving models of how amygdalar circuits contribute to cognition and mental health.

## Teaser

Specialized cell types and links to psychiatric disorders are revealed by genetic mapping of primate amygdala neurons.

## INTRODUCTION

The amygdala plays a central role in emotional learning and memory (*1*, *2*), and is implicated in a number of psychiatric disorders, most notably those associated with stress and substance use (*3*, *4*). In primates, the amygdala is a large, complex structure composed of numerous subnuclei that contain a diverse array of heterogeneous and highly specialized cell types (*5*). Understanding the anatomical organization and evolutionary conservation of the primate amygdala at cellular resolution is essential for uncovering the neural mechanisms that underlie the regulation of emotional memories and contribute to the development of psychiatric disorders (*6*). However, the cellular and molecular identity of these populations and the extent to which these identities are conserved across species remain unclear. This limitation hinders our ability to identify common and distinct subtypes across the major subdivisions and nuclei of the primate amygdala.

Most amygdala neurons are located in the lateral nucleus (LA), basal nucleus (BA), and accessory basal nucleus (aBA) divisions of the basolateral amygdala (BLA) complex, the central nucleus (CeA), and the intercalated cell masses (ITCs) (*7*, *8*). The BLA complex integrates sensory input, assigns hedonic valence, and is largely composed of excitatory glutamatergic neurons that project both within and outside of the amygdala (*9*), as well as a diverse array of GABAergic inhibitory neurons comprising complex microcircuitry (*10*). In contrast, the CeA functions as a major output hub, translating emotionally salient information into behavioral responses via projections to the hypothalamus and brainstem, and is composed entirely of GABAergic inhibitory neurons (*11*). The ITCs form a web with densely packed islands of GABAergic neurons that divide the BLA and CeA along the anterior-posterior axis in primates. In rodents, the ITCs play key roles in gating information flow within the amygdala and are implicated in fear regulation (*12*). Across both rodents and primates, these subdivisions are composed of neuronal populations with various developmental origins (*13*). For example, the CeA and ITC inhibitory neurons are derived from the lateral ganglionic eminence (LGE) (*12*, *14*), whereas inhibitory BLA neurons originate from either the medial ganglionic eminence (MGE) and caudal ganglionic eminence (CGE) (*15*). While anatomical and electrophysiological investigations in rodents revealed the existence of distinct glutamatergic populations within the BLA complex (*16*), distinct excitatory populations remain to be identified in primates.

Recent advances in single-nucleus RNA sequencing (snRNA-seq) and spatial transcriptomics have revolutionized the study of molecular and cell type diversity in the brain, enabling comprehensive identification of transcriptionally distinct neuronal subtypes at unprecedented resolution. In rodents, snRNA-seq and spatial transcriptomics have begun to identify subregion-specific neuron types that are molecularly and functionally distinct (*17*, *18*). Similarly, researchers have begun to profile the postmortem human amygdala using snRNA-seq to identify cell types implicated in psychiatric disorders (*19*). Although studies in marmosets (*20*), macaques (*21*, *22*), and humans (*22*–*24*) have begun to catalog neuronal diversity in primates, these studies have largely lacked anatomical resolution. As a result, it remains difficult to map transcriptionally defined cell populations onto specific amygdala subdivisions, hindering efforts to link gene-specific function or psychiatric disease risk to specific neural circuit components. The development of a spatially informed cell type atlas in primates would provide a bridge for integrating preclinical and clinical studies, allowing researchers to more directly translate rodent findings onto homologous structures in the primate brain to better infer the functional roles of conserved cell types across species.

To address this gap, we used a tissue sampling strategy that targeted each of the major amygdala subdivisions in macaques, baboons, and humans coupled with cross-species integration to construct an snRNA-seq atlas that delineates subdivision-specific amygdala neuron types across primate species. We identified five broad classes of excitatory neurons, as well as heterogeneous inhibitory populations of MGE, CGE, and LGE origins. Both excitatory and inhibitory neuron types display substantial spatial variation across the primate amygdala. Using these data, we found robust subdivision-specific excitatory marker genes that are conserved across humans and nonhuman primates (NHPs) and validated these findings using fluorescence in situ hybridization (FISH). We also quantified the extent to which these neuron types overlap with neuron types identified in rodents, as well as other NHP and human datasets. Last, we use both gene set and genetic enrichment analyses to assess the potential cell type–specific enrichment of psychiatric disease risk. These data demonstrate the intricate neuronal organization of the primate amygdala and provide a foundational resource for understanding its functional architecture and relevance to psychiatric disorders. Coupled with existing molecular genetic tools, information in this atlas will enable perturbation studies that selectively target glutamatergic and GABAergic function in specific subdivisions in the primate amygdala to determine their role in motivated behaviors (*25*). Moreover, as anatomical mapping tools mature in the NHP primate, this atlas can link molecularly distinct neuron types to axonal projection anatomy, similar to approaches in rodents (*18*). To facilitate these goals and promote the use of these data by the broader neuroscience community, all data and code are freely accessible, and we created cloud-based tools that enable data exploration (see Materials and Methods).

## RESULTS

### Precise subdivision sampling and snRNA-seq data generation of the rhesus macaque, baboon, and human amygdalae

**Fig. 1.**
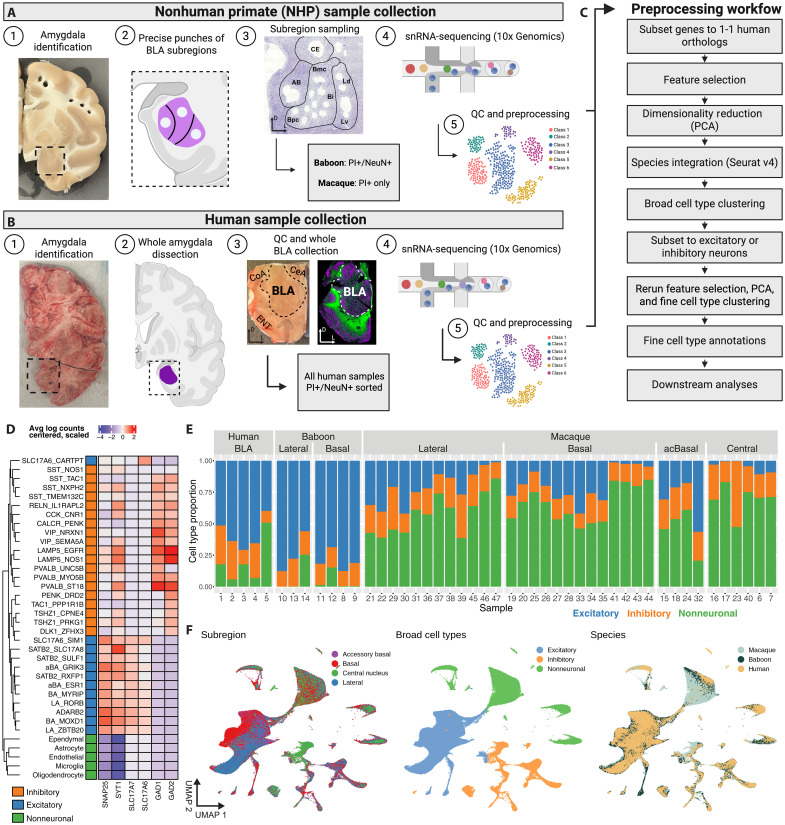
snRNA-seq combined with targeted mesoscale sampling to broadly characterize and spatially localize cell types in the amygdala of humans, baboons, and macaques. (**A** and **B**) Visualization of targeted mesoscale sampling of the amygdala in humans and NHPs (*M. mulatta* and *P. anubis*). The entire basolateral complex in humans including the aBA, BA, and LA was collected from fresh frozen tissue sections, and all samples were then enriched for neurons using NeuN+ staining (81.9% neurons in total). In macaques, targeted sampling used biopsy punches of fresh, unfrozen brain slabs to sample the BA, aBA, LA, and CeA. In baboons, the BA and LA were sampled, and all samples were then enriched for neurons using NeuN+ staining (80 to 99% neurons in total). QC, quality control. (**C**) Illustration of the preprocessing workflow to identify cell types across all three species. (**D**) Heatmap of gene expression for broad marker genes used to characterize cell type clusters as excitatory neurons, inhibitory neurons, or nonneuronal cells. Dendrogram ordering of fine cell types (rows) was conducted via hierarchical clustering of pseudobulked gene expression profiles. (**E**) Stacked bar plots depicting the proportion of broad cell types per sample, grouped by species and the amygdala nuclei that were sampled. (**F**) UMAP visualizations after integration across species of sequenced nuclei colored by amygdala subdivision (left), broad cell type annotations (middle), and fine cell type annotations (right). CE, CeA; Ld, dorsal region of the LA; Lv, ventral region of the LA; Bmc, magnocellular region of the BA, Bi, intermediate region of the BA; Bpc, parvocellular region of the BA; AB, aBA. (A) to (C) Created in BioRender. M. Lab, (2025) https://BioRender.com/x86b098.

We acquired postmortem tissue containing the amygdala from five rhesus macaques (ages 5 to 18 years, *M* = 11.85; three females), two female olive baboons (ages 3 to 4 years, *M* = 3.97; table S1), and five human brain donors (ages 34.5 to 70.64 years, *M* = 50.27; one female; table S2). To sample individual nuclei of the amygdaloid complex, we collected targeted tissue punches (1 to 2.5 mm in diameter) of the aBA, BA, LA, and CeA (Fig. 1A) in macaques and baboons. In humans, tissue from the entire BLA complex was collected in multiple 100-μm sections from a tissue block containing all major nuclear divisions (Fig. 1B).

We used the 10x Genomics Chromium platform to generate snRNA-seq on the collected NHP and human samples. Quality control (QC) was independently performed for each of the 47 samples (figs. S2 to S4), and preprocessing followed a standard workflow (Fig. 1C), resulting in a final dataset of 131,812 nuclei [>700 unique molecular identifiers (UMIs) per library; mean, 11,179.56 UMIs]. To identify conserved cell types across species without overcorrecting for potential species differences, we performed data integration (see Materials and Methods) across subjects (both humans and NHPs) rather than directly integrating across species. This strategy resulted in strong mixing of both species and subjects across all clusters (Fig. 1F and figs. S5 and S6). We visualized these data using uniform manifold approximation and projection (UMAP), a dimensionality reduction technique that projects high-dimensional single-cell data into two dimensions. Notably, we observed clear dissociation between major amygdala subdivisions in the overall inhibitory and excitatory neuron clusters (Fig. 1F). We then identified broad cell types using canonical marker genes for neuronal (excitatory and inhibitory) and nonneuronal cell types (Fig. 1, D and F). There was a clear dissociation between the CeA and the other major nuclei in the overall ratio of GABAergic to glutamatergic neuron types. Of the neurons that came from tissue punches of the CeA, 79.6% were GABAergic, whereas neuronal populations derived from punches of the other subdivisions contained at most 40% GABAergic neurons. Glutamatergic neurons were more prevalent in the aBA (59.9%), LA (68.7%), and BA (70.15%). Although the CeA samples displayed ~20% excitatory neurons, nearly all of these excitatory populations were derived from a few macaque samples that likely captured the neighboring aBA, BA, LA, or medial nuclei because of the small size and proximity of the CeA—especially its rostral extent (Fig. 1E). This underscores the challenges in achieving precise dissections of amygdalar subdivisions using fresh tissue. Nonetheless, we observed notably consistent cell type proportion differences across amygdala subdivisions for both broad (Fig. 1, E and F) and fine cell types (figs. S5 and S6).

To delve further into how specific subtypes of GABAergic and glutamatergic neurons were distributed across the four major nuclei, we subset glutamatergic and GABAergic neurons from other cell types into two independent datasets. We then repeated feature selection, dimensionality reduction, dataset integration, clustering, and cell type annotation to derive fine cell types. Overclustering was avoided using the pairwise modularity ratio of observed to expected edge weights to ensure that all clusters were well separated. This analysis identified 19 types of GABAergic neurons and 13 types of glutamatergic neurons (Fig. 1D). For both inhibitory (fig. S5) and excitatory neurons (fig. S6), when we examined the frequency of each cell type within each sample organized by species and location, we observed that all cell types were present in all three species (Fig. 1E). All fine cell type clusters displayed high numbers of genes detected and total library size (fig. S7), with neurons naturally containing higher gene counts than nonneurons.

### Molecular diversity and cross-species conservation of inhibitory neurons

**Fig. 2.**
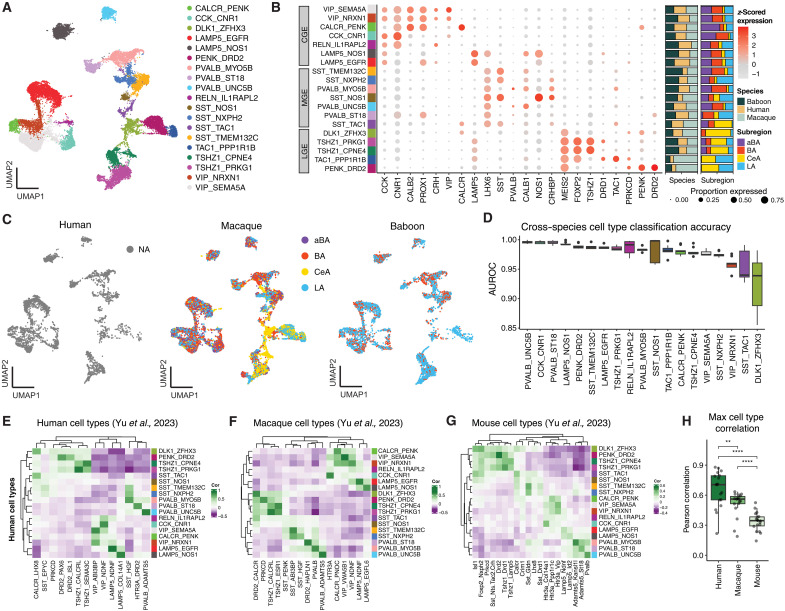
Transcriptomic profiling and cross-species comparisons of inhibitory neurons in the primate amygdala. (**A**) UMAP embedding of the integrated snRNA-seq dataset of amygdala inhibitory cell types across primates species, colored by 19 molecularly defined cell type clusters (see legend). (**B**) Dot plot summarizing the scaled expression (color) and percentage of cells expressing (size) for key marker genes across annotated cell types; hierarchical dendrograms derived from the clustering of marker expression. (**C**) Same UMAP embedding from (A) split by species and colored by annotation dissection location, displaying cell type heterogeneity across subdivisions. Nuclei sampled from humans are colored gray (NA) because the entire BLA area was sampled rather than precise subdivisions. (**D**) Cross-species cell type classification performance (AUROC) calculated by unsupervised MetaNeighbor analysis, demonstrating the high replicability of molecular signatures across species. (**E** to **G**) Pearson correlation heatmaps comparing the top 100 marker genes per cell type between our human dataset and Yu *et al.* (*22*) human (E), macaque (F), and mouse (G) amygdala datasets (*22*), highlighting strong one-to-one mapping to human and macaque datasets and weaker mapping to mouse cell types. (**H**) Maximum cell type correlations for each of our human cell types compared to Yu *et al.* (*22*) datasets. **P < 0.01 and ****P < 0.0001.

The amygdala is known to contain a large diversity of GABAergic inhibitory neurons, with the BLA containing a mix of MGE- and CGE-derived neurons and the CeA and ITCs largely comprising LGE-derived neurons. In line with this, we identified 19 transcriptionally distinct inhibitory neuron types spanning these major ganglionic eminence lineages (Fig. 2, A and B) that displayed a wide diversity of neuromodulatory receptor expression (fig. S8). Each cell type cluster was annotated using a combination of canonical marker genes and top-ranked marker genes identified by one-versus-all differential expression analysis. We identified seven CGE-derived inhibitory clusters expressing either *CCK*, *VIP*, or *LAMP5*; seven MGE-derived inhibitory clusters expressing either *SST* or *PVALB*; and five LGE-derived clusters expressing canonical CeA markers *PRKCD* and *DLK1*, as well as the canonical ITC marker *TSHZ1.* All of these inhibitory cell types were sampled at similar proportions across primate species, with the exception of LGE-derived neurons, which were disproportionately represented in macaques because of targeted sampling of the CeA in that species (Fig. 2, B and C). We found nearly identical cell type clusters when we performed our clustering pipeline within species followed by cross-species label transfer, demonstrating that our integration approach preserved the biologically meaningful structure and did not obscure potential species-specific cell types (fig. S9). In addition, all cell types showed high cross-species classification accuracy [area under the receiver operating characteristic (AUROC) > 0.8; fig. S10], indicating strong evolutionary conservation (Fig. 2D).

To compare our cell type clusters to prior work and further assess the evolutionary conservation of inhibitory neurons, we compared our human inhibitory cell type dataset to the human, macaque, and mouse datasets previously published by Yu *et al.* (*22*). For each of our cell types, we computed the Pearson correlation of gene expression using the top 100 human marker genes from our dataset (Fig. 2, E to G). This analysis revealed a clear one-to-one correspondence between our human inhibitory cell types and those in both the Yu *et al.* (*22*) human and macaque datasets. In contrast, correspondence dropped substantially in the mouse dataset, with few clear one-to-one pairs. In line with this, the maximum correlation of cell type mappings decreased with evolutionary distance across human-human, human-macaque, and human-mouse comparisons (Fig. 2H). We found similar results when we compared our macaque and baboon datasets to the Yu *et al.* (*22*) datasets (fig. S11). This highlights the high degree of molecular conservation among primate inhibitory neurons while also underscoring the transcriptomic divergence of homologous populations in rodents.

### Subnuclear organization of inhibitory neuron types

Although a prior study successfully characterized and cataloged the inhibitory populations found in the primate amygdala (*10*), it largely relied on bulk sampling strategies that indiscriminately profile the entire amygdala. As a result, the subdivision-specific specialization of inhibitory cell types remains largely unknown. To overcome this limitation, we used our anatomically targeted dissections of the LA (*n* = 15), BA (*n* = 17), aBA (*n* = 4), and CeA (*n* = 6) in NHPs, enabling precise compositional comparisons across subnuclei. To quantify differences in cell type composition between regions, we used a linear mixed-effects modeling framework (see Materials and Methods) (*26*). First, we applied the well-established centered log ratio (CLR) transformation to the cell type proportions, which accounts for the fixed sum constraint and nonnormality inherent to compositional data. We then used precision-weighted linear mixed models that included sex, species, and subdivision as fixed effects with subject identity modeled as a random effect to account for repeated measures. The precision weights act to reduce the influence of outlier samples that may have been off-target or contaminated with cell types from neighboring subdivisions. Additional linear contrasts were fit for each pairwise comparison of interest. This framework allows for robust inference of regional differences in inhibitory neuron composition while appropriately handling both the compositional nature and repeated measure design of the data.

**Fig. 3.**
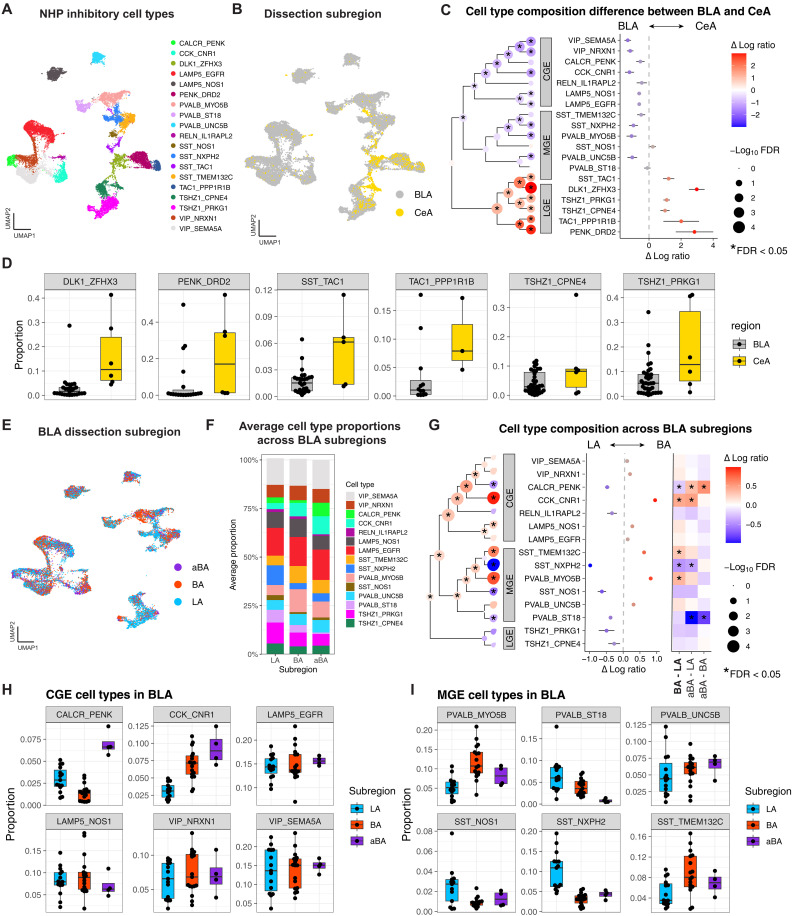
Compositional analysis reveals distinct inhibitory neuron populations in the CeA and subdivision-specific patterns across the BLA. (**A**) UMAP projection of inhibitory neurons colored by fine cell type. (**B**) Same UMAP colored by dissection subdivision. (**C**) Visualization of cell type compositional analysis using linear mixed models: (left) dendrogram hierarchically clustered on the basis of gene expression; (right) estimated effect size from the regression analysis of cell type composition associated with the CeA (*n* = 6) versus BLA (*n* = 36). The color indicates effect size, and * indicates FDR < 0.05. (**D**) Box plots of selected CeA-enriched cell types showing elevated proportions in the CeA versus BLA across samples. (**E**) Schematic overview of approach to refine compositional analysis within BLA-only samples, excluding CeA punches and CeA-specific cell types. (**F**) UMAP projection of remaining inhibitory neurons colored by subdivision after CeA removal. (**G**) Visualization of cell type compositional analysis using linear mixed models: (left) dendrogram hierarchically clustered on the basis of gene expression; (middle) estimated effect size from the regression analysis of cell type composition associated with the BA versus LA; (right) heatmap displaying effect size estimates of cell type compositions across pairwise comparisons of BLA subdivisions. The color indicates effect size, and * indicates FDR < 0.05. (**H** and **I**) Box plots of BLA proportions for representative CGE-derived (H) and MGE-derived (I) inhibitory cell types across the LA, BA, and aBA.

Using this framework, we first verified that the BLA and CeA are enriched for inhibitory neurons that expressed marker genes for MGE/CGE and LGE domains, respectively. To test this, we collapsed all LA, BA, and aBA samples into a single “BLA” group (*n* = 36) and compared their cell type composition to the CeA punches (*n* = 6). The inhibitory cell types and their modified anatomical dissection location can be viewed in the UMAP space in Fig. 3 (A and B). Applying our compositional model to the BLA versus CeA comparison, we identified numerous cell types with significant regional biases [all *P* values adjusted for the false discovery rate (FDR) < 0.05; Fig. 3C]. As expected, most MGE- and CGE-derived populations were preferentially enriched in the BLA. This includes both *VIP*^+^ populations, both *LAMP5*^+^ populations, CCK_CNR1, two *PVALB*^+^ populations (PVALB_MYO5B and PVALB_UNC5B), and SST_NXPH2 (FDR < 0.05). In contrast, all five LGE-derived populations including DLK1_ZFHX3, both *PRKCD*^+^ populations (PENK_DRD2 and TAC1_PPP1R1B), both *TSHZ1*^+^-intercalated populations, and the MGE-derived SST_TAC1 population were enriched in CeA punches (FDR < 0.05). This directly aligns with prior work demonstrating that *SST*^+^/*TAC1*^+^ and *PRKCD*^+^ inhibitory neurons are unique to the CeA (*27*). *TSHZ1*^+^-intercalated cells are not part of the CeA itself but rather form anatomically distinct clusters that surround the boundaries of both the CeA and BLA. As such, their enrichment in CeA-targeted dissections reflects their proximity to CeA rather than inclusion within the CeA proper. To further visualize these differences, we plotted the untransformed proportions of CeA-enriched cell types across samples (Fig. 3D). In each case, these populations show markedly higher abundance in the CeA dissections compared to the BLA, consistent with their anatomical localization and development origin. These results highlight the power of our mixed modeling framework and spatially resolved sampling strategy to uncover meaningful, subdivision-specific distinctions in cell type composition.

After establishing broad differences in inhibitory neuron composition between BLA and CeA, we next asked whether any MGE or CGE cell types exhibit subdivision-specific patterns within the BLA itself. To test this, we excluded all CeA samples and CeA-enriched cell types identified above to avoid potential bias from off-target sampling or low-level contamination in BLA punches. We did, however, retain the *TSHZ1*^+^ cell types. Because ITCs are interspersed between BLA subnuclei, we aimed to determine whether these populations could be localized to distinct subdivisions. UMAP visualization of the retained BLA samples (Fig. 3F) and cell type distribution bar plots (Fig. 3F) revealed moderate heterogeneity across subdivisions. We then compared the composition of inhibitory neurons across the subnuclei of the LA (*n* = 15), BA (*n* = 17), and aBA (*n* = 4) using the sample mixed modeling framework described above. To identify specific subdivision-enriched populations, we fit pairwise linear contrasts for each cell type comparing BA versus LA, aBA versus LA, and aBA versus BA (Fig. 3G). Compositional analysis revealed several CGE- and MGE-derived populations with significant compositional differences across LA, BA, and aBA (all FDR < 0.05). As shown in Fig. 3 (G and H), we identified that CGE-derived CALCR_PENK neurons are enriched in aBA relative to both LA and BA (FDR < 0.05), while CCK_CNR1 neurons are enriched in both aBA and BA compared to the LA (FDR < 0.05). Conversely, both populations of *LAMP5*^+^ and *VIP*^+^ neurons were equally distributed across the BLA subnuclei. For MGE-derived neurons, we found that distinct populations of *SST*^+^ and *PVALB*^+^ neurons are enriched in different BLA nuclei. Specifically, SST_NXPH2 is enriched in the LA relative to BA and aBA (FDR < 0.05), whereas SST_TMEM132C showed the opposite trend, with higher abundance in the BA relative to the LA (FDR < 0.05; Fig. 3, G and I). Among *PVALB*^+^ populations, PVALB_ST18 is enriched in both the LA and BA relative to the aBA (FDR < 0.05), whereas PVALB_MYO5B was more abundant in the BA relative to the LA (FDR < 0.05). In contrast, SST_NOS1, putative long-range *SST*^+^ projection neurons (*28*), and PVALB_UNC5B were found to be equally distributed across BLA subnuclei (Fig. 3, G and I).

We initially hypothesized that the spatial localization of ITCs might reveal medial-lateral differences across BLA subnuclei. However, we did not observe significant variation in the abundance of either *TSHZ1*^+^ ITC across LA, BA, or aBA punches. To support future efforts in identifying and spatially localizing ITCs and other spatially distinct inhibitory neurons, we conducted cross-species correlation analyses of one-versus-all marker gene t-statistics (see Materials and Methods) to identify cell type marker genes (fig. S12) that were highly conserved between humans and monkeys. For *TSHZ1*^+^ ITCs, we found that both *CPNE4* and *PRKG1* were among the highly conserved marker genes for TSHZ1_CPNE4 and TSHZ1_PRKG1, respectively (fig. S12, A and B). For *SST*^+^ populations, we found that *NPY* was the top conserved marker gene for BA-enriched SST_TMEM132C neurons, while *PRKCG* was the top conserved marker gene for LA-enriched SST_NXPH2 neurons (fig. S12, D and E). For *PVALB*^+^ neurons, we found that ST18 was the top conserved marker gene for LA/BA–enriched PVALB_ST18 neurons, whereas *MYO5B* was the top marker for BA-enriched PVALB_MYO5B neurons (fig. S12, G and H).

### Molecular diversity and cross-species conservation of excitatory neurons

**Fig. 4.**
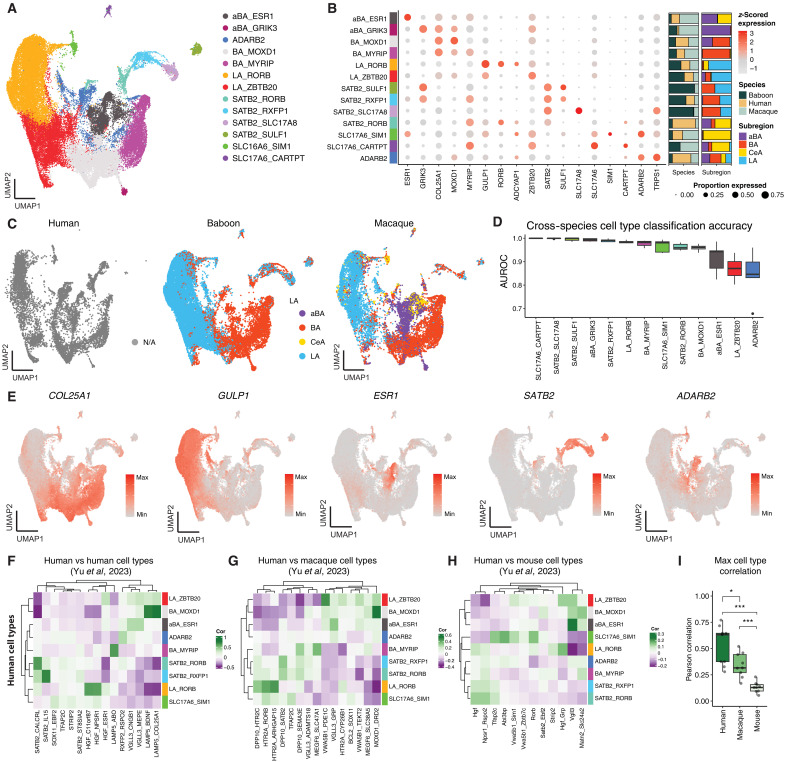
Transcriptomic profiling and cross-species comparisons of excitatory neurons in primate amygdala. (**A**) UMAP embedding of the integrated single-nucleus dataset of amygdala excitatory cell types across primate species, colored by 13 molecularly defined cell type clusters (see legend). (**B**) Dot plot summarizing the scaled expression (color) and percentage of cells expressing (size) for key marker genes across annotated cell types; hierarchical dendrograms derived from the clustering of marker expression. (**C**) Same UMAP embedding from (A) split by species and colored by annotation dissection location, displaying cell type heterogeneity across subdivisions. Nuclei sampled from humans are colored gray (NA) because the entire BLA area was sampled rather than precise subdivisions. (**D**) Cross-species cell type classification performance (AUROC) calculated by unsupervised MetaNeighbor analysis, demonstrating the high replicability of molecular signatures across species. (**E**) UMAP visualization of top marker genes for BA (*COL25A1*), LA (*GULP1*), aBA (*ESR1*), SATB1 (*SATB1*), and ADARB2 (*ADARB2*) cell types. (**F** to **H**) Pearson correlation heatmaps comparing the top 100 marker genes per cell type between our human dataset and Yu *et al.* (*22*) human (F), macaque (G), and mouse (H) amygdala datasets, highlighting strong one-to-one mapping to human and macaque datasets and weaker mapping to mouse cell types. (**I**) Maximum cell type correlations for each of our human cell types compared to Yu *et al.* (*22*) datasets. *P < 0.05 and ***P < 0.001.

Compared to GABAergic neurons, clear hypotheses about the number of glutamatergic neuron types and their spatial localization in the primate amygdala are lacking. While previous snRNA-seq studies have successfully identified various groups of molecularly distinct excitatory neuron types, they were unable to determine subdivision specificity because of bulk sampling strategies. Similarly, prior neuroanatomical studies noted that glutamatergic neurons differ in terms of their size (e.g., magnocellular versus parvocellular subdivisions particularly in the BA) and excitability (*5*, *16*) but have not identified neuron types specific to nuclear subdivisions. Here, we identified 13 distinct types of glutamatergic neurons in the primate amygdala across all three primate species using our cross-species integration strategy (Fig. 3, A and B). Similar cross-species mapping was found using the complementary within-species clustering and label transfer approach described above (fig. S13). UMAP visualization of these clusters colored by anatomical dissection location in monkeys revealed that several major excitatory populations were almost exclusively sampled from a single BLA subdivision, suggesting highly localized spatial identities (Fig. 4C). We classified these 13 populations into five major groups on the basis of either their anatomical subdivision origins or molecular identity: LA neurons, BA neurons, aBA neurons, *SATB2*^+^ neurons, vGlut2 (*SLC17A6*^+^) neurons, and a small population of *ADARB2^+^*/*SLC17A7^+^* neurons (Fig. 4E and fig. S14). We found that all subdivision-specific cell types were equally sampled across species, except for aBA neurons, which were disproportionality represented in humans and macaques as the aBA was not directly sampled in baboons (Fig. 4B). However, we note substantial species differences in the *SATB2*^+^ and *SLC17A6*^+^ populations. The SATB2_RORB population was disproportionately more abundant in humans, whereas the SATB2_SULF1, SATB2_SLC17A8, and SLC17A6_CARTPT populations were primarily sampled in monkeys. Despite this, the cross-species classification accuracy was high (AUROC > 0.8) for all excitatory cell types (Fig. 4D and fig. S15), demonstrating that the molecular identities of these neurons are strongly conserved.

To further contextualize these excitatory neuron types, we compared our excitatory types to the human, macaque, and mouse datasets reported by Yu *et al.* (*22*). Using the same marker gene correlation strategy described for inhibitory neurons, we observed clear relationships between many of our human excitatory neurons and human cell types identified by Yu *et al.* (*22*). We found that our LA, BA, and aBA cell types generally map onto their *HGF^+^* (*HTR2A^+^* in macaques), *LAMP5^+^*, and *VGLL3^+^* populations, respectively. At finer resolution, we observed one-to-one or one-to-many mapping between cell types. For example, our BA_MYRIP and BA_MOXD1 cell types respectively mapped onto their human *LAMP5^+^* populations expressing *ABO*^+^ and *BDNF*^+^/*COL25A1*^+^. We also observed strong one-to-one mapping of *SATB2^+^* populations across human datasets. However, there was a *SATB2*^+^ population present in the Yu *et al.* (*22*) human dataset, SATB2_STBSIA2, that did not have a clear match in our human dataset. Instead, this population mapped to the SATB2_SLC17A8 populations present in our NHP datasets (fig. S16). This suggests that SATB2_SLC17A8 is not a monkey-specific neuron type but rather was not sampled in our human dataset. In contrast, SATB2_SULF1 only displayed moderate mapping between our and Yu *et al*.’s (*22*) NHP dataset. Last, our *ADARB2^+^* cell type did not show clear mapping to any of the Yu *et al.* (*22*) datasets and was primarily sampled from a single human donor (Br8331; fig. S6C), suggesting that it may represent a nonamygdalar cell type. Similar to inhibitory neurons, the average correlation between top cell type mappings decreased across human-human, human-macaque, and human-mouse comparisons (fig. S12B). In summary, our excitatory neuron types broadly align with those reported by Yu *et al.* (*22*) and largely recapitulate their cross-species comparisons. Using our subnuclear sampling strategy, we have extended these findings by localizing their *HGF*^+^, *LAMP5*^+^, and *VGLL3*^+^ cell types to specific amygdala subdivisions.

### Multiple types of excitatory neurons are organized by subdivision

**Fig. 5.**
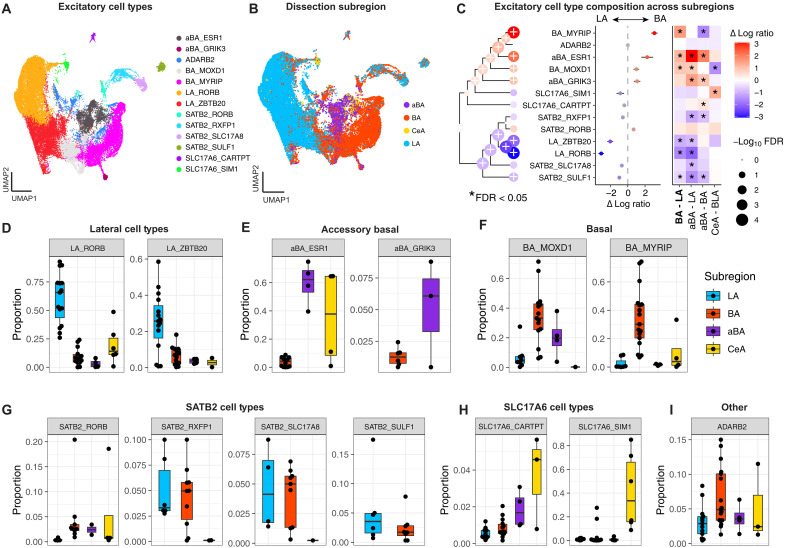
Subdivision-specific composition of excitatory neuron subtypes in the primate amygdala. (**A**) UMAP visualization of annotated excitatory neuron subtypes identified from macaque donors. (**B**) Same UMAP as in (A), colored by manually annotated subdivision of origin: LA, BA, aBA, and CeA. (**C**) Visualization of cell type compositional analysis using linear mixed models: (left) dendrogram hierarchically clustered on the basis of gene expression; (middle) estimated effect size from the regression analysis of cell type composition associated with the BA versus LA; (right) heatmap displaying effect size estimates of cell type compositions across pairwise comparisons of BLA subdivisions. The color indicates effect size, and * indicates FDR < 5%. (**D** to **I**) Box plots showing the proportion of individual excitatory subtypes per sample across subdivisions. Subtypes are grouped by anatomical enrichment: (D) LA-enriched (e.g., LA_RORB and LAZBTB20), (E) aBA-enriched (e.g., aBA_ESR1 and aBA_GRIK3), (F) BA-enriched (e.g., BA_MOXD1 and BA_MYRIP), (G) *SATB2*^+^, and (H) *SLC17A6*^+^ subtypes. (I) Additional subtypes with distinct profiles (*ADARB2*).

Our initial UMAP visualization revealed that several excitatory neuron populations were almost exclusively sampled from individual BLA subdivisions (Fig. 5, A and B). To formally test this, and to assess whether additional excitatory cell types also exhibit subdivision-specific enrichment, we applied the same compositional modeling framework previously described for inhibitory neurons. We once again accounted for potential sex and species differences by including them as covariates in the model, with individual subjects modeled as a random effect to handle repeated measures. We performed pairwise contrasts between LA, BA, and aBA to quantify subdivision enrichment across the 13 excitatory neuron types.

We confirmed that our putative LA-specific cell types (LA_RORB and LA_ZBTB20) were enriched in the LA compared to both the BA and aBA (Fig. 5, C and D), BA-specific cell types (BA_MYRIP and BA_MOXD1) were enriched in the BA compared to the LA (Fig. 5, C and F), and aBA-specific cell types (aBA_ESR1 and aBA_GRIK3) were enriched in the aBA compared to the LA and BA (all FDR < 0.05; Fig. 5, C and E). Three of the *SATB2*^+^ populations were exclusively sampled from BA and LA punches (FDR < 0.05), whereas SATB2_RORB was equally distributed across BLA subdivisions (Fig. 5, C and G). Last, we noted that our *SLC17A6*^+^ populations were more frequently sampled from dorsal regions such as the aBA and CeA (Fig. 5, C and H), and the *ADARB2*^+^ populations did not differ by subdivision (Fig. 5, C and I). To confirm this, we performed a linear contrast comparing the composition of CeA punches to the average composition of BLA punches (CeA versus BLA; Fig. 5C). We found that SLC17A6_SIM1 neurons originated almost entirely from either CeA punches (FDR < 0.05) or dorsal BA and LA punches (FDR < 0.05; fig. S17). Given that the CeA is a purely GABAergic structure, it is most likely that this excitatory population originates from the neighboring medial amygdala (MeA), consistent with prior reports showing that *SIM1*^+^ neurons are selectively localized to the MeA in primates (*20*). Although SLC17A6_CARTPT neurons were not significantly enriched in the CeA compared to the BLA, *CARTPT* is an established MeA and CeA marker gene in rodents. We speculate that this population also originates from the MeA.

### Conserved excitatory marker genes delineate BLA subdivisions

**Fig. 6.**
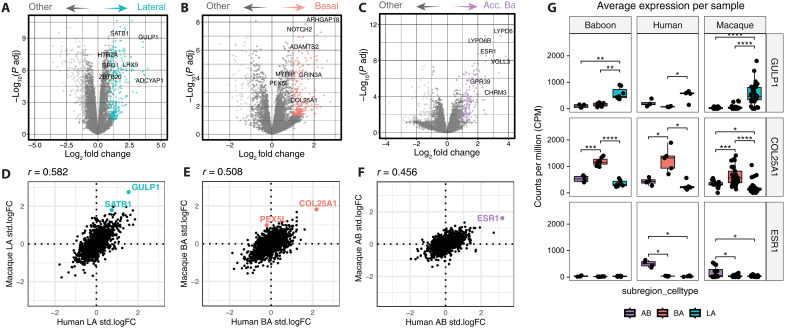
Pseudobulk differential expression analysis of LA versus BA excitatory neurons reveals conserved marker genes and molecular function. (**A** to **C**) Volcano plots showing differentially expressed genes between excitatory neurons from each BLA subdivision (LA, BA, and aBA) compared to the other two subdivisions in NHPs. Top candidate marker genes are highlighted for each subdivision. (**D** to **F**) Scatterplots showing cross-species conservation of log_2_FC values for each subdivision, comparing standardized pseudobulk expression in human versus macaque. (**G**) Box plots showing regularized log counts per million (CPM) values per sample for GULP1, COL25A1, and ESR1—the top marker genes for LA, BA, and aBA, respectively—across species and subdivisions. Data points represent individual pseudobulk samples. Box plots display the median with upper and lower quartiles. *P < 0.05, **P < 0.01, ***P < 0.001, and ****P < 0.0001.

Having identified multiple neuron types that were spatially localized to specific BLA subdivisions, we next identified marker genes for excitatory neurons across subdivisions. To do so, we grouped neuron types by their anatomical origins and conducted pseudobulk differential expression analysis by aggregating UMI counts from excitatory neurons within each LA (*n* = 15), BA (*n* = 17), and aBA (*n* = 4) sample from NHPs to identify genes enriched in excitatory neurons sampled from the respective subdivisions (see Materials and Methods for more details). This approach avoids inflated statistical significance by using each sample, rather than individual cells, as independent observations. Similar to our compositional analyses, we used the dream linear mixed model framework that rigorously accounts for random effects and covariates (*29*). For each subdivision, we performed linear contrast to compare one nucleus against the mean of the other two nuclei (e.g., LA versus the average of BA and aBA) to identify subdivision-specific gene expression (Fig. 6, A to C). This analysis revealed 296 genes that were up-regulated in LA excitatory neurons (FDR < 0.05, log fold change (logFC) > 1; Fig. 6A), 233 genes up-regulated in BA excitatory neurons (FDR < 0.05, logFC > 1; Fig. 6B), and 148 up-regulated genes in aBA excitatory neurons (FDR < 0.05, logFC > 1; Fig. 6C). In these differentially expressed gene sets, the previously identified *GULP1*, *COL25A1*, and *ESR1* genes were among the top marker genes for excitatory neurons in the LA, BA, and aBA, respectively, of NHPs. Of note, we also observed that *VGLL3* was one of the top marker genes for aBA cells (Fig. 6C), further confirming our spatial mapping between primates and rodents using the Yu *et al.* (*22*) data. We next investigated whether these division-specific marker genes are conserved in the human amygdala. To test this, we first collapsed the fine cell type clusters derived largely from the LA (LA_ZBTB20 and LA_RORB), BA (BA_MYRIP and BA_MOXD1), and aBA (aBA_GRIK1 and aBA_ESR1) into putative subdivisional superclusters. Within species, we then performed single-cell differential expression analysis; found species-specific marker genes for putative LA, BA, and aBA excitatory superclusters; and correlated the logFC to find conserved excitatory marker genes for the LA (Fig. 6D), BA (Fig. 6E), and aBA (Fig. 4F). This successfully identified *GULP1*, *COL25A1*, and *ESR1* as the top conserved markers across primate species.

**Fig. 7.**
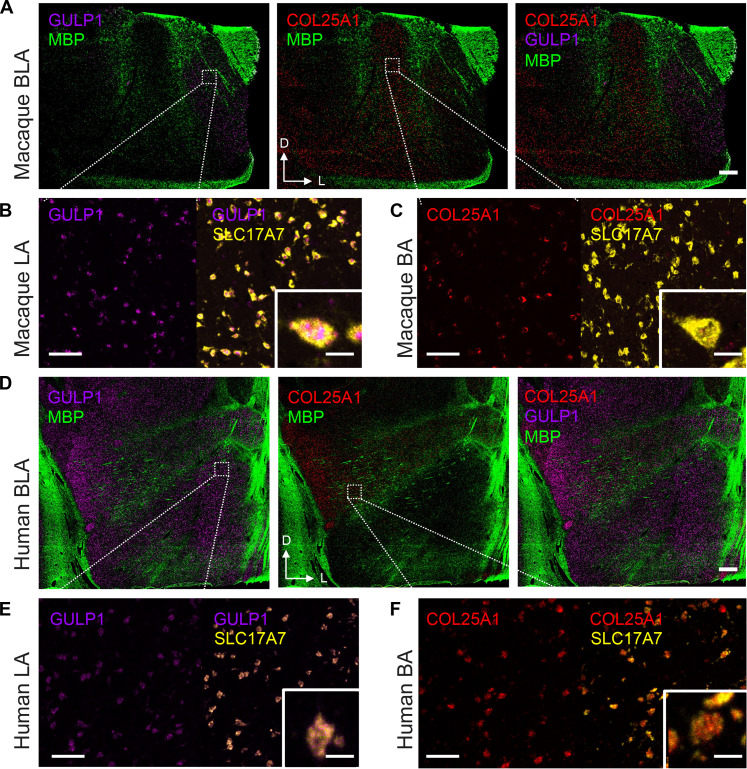
Marker gene expression for macaque and human LA amygdala and BA amygdala. 2× smFISH images of the basolateral region of the macaque (**A**) and human (**D**) brains illustrating the expression of *GULP1* (magenta) in LA and *COL25A1* (red) in BA. The *MBP* signal (green) represents white matter for anatomical landmarks. Dorsal (D) and lateral (L) arrows are added for tissue directionality. White boxes represent approximate locations of zoomed-in images. Scale bars, 1000 μm. Zoomed-in smFISH images illustrating the coexpression of *GULP1* (magenta) and *SLC17A7* (yellow) within the 20× macaque (**B**) and 40× human (**E**) LA. Scale bars, 100 μm. The inset illustrates a representative neuron coexpressing *GULP1* and *SLC17A7*. Scale bars of the insets, 10 μm. Zoomed-in smFISH images illustrating the coexpression of *COL25A1* (red) and *SLC17A7* (yellow) within the 20× macaque (**C**) and 40× human (**F**) BA. Scale bars, 100 μm. The inset illustrates a representative neuron coexpressing *COL25A1* and *SLC17A7*. Scale bars of the insets, 10 μm.

To further establish these genes as high-confidence markers of spatially distinct glutamatergic cell types across primate species, we assessed spatial patterns of *GULP1* and *COL25A1* expression among excitatory neurons coexpressing *SLC17A7* using single-molecule FISH (smFISH) in tissue from independent macaques (*n* = 2; one female) and a human donor (*n* = 1; one male) (fig. S18). As predicted from our cross-species transcriptomic profiling using tissue punch annotations, there was no overlap in the spatial expression of these marker genes in the macaque (Fig. 7 and fig. S19). Using myelin basic protein (*MBP*) gene expression to define boundaries between the major amygdala nuclei, we found that *GULP1* expression was confined to the LA, whereas *COL25A1* expression was present throughout all subdivisions of the BA and also present in the aBA (Fig. 7, A to C, and fig. S19). In humans, we similarly found that *COL25A1* expression was restricted to the BA and that *GULP1* expression predominated in the LA (Fig. 7, D to F). Compared to macaques, we did observe more spatial overlap in the gross expression of *GULP1* and *COL25A1* in parts of the BA in humans, but we did not observe the coexpression of these two genes within individual *SLC17A7^+^* nuclei. This is somewhat expected (i.e., neither cluster of sequenced, excitatory neurons was absolutely derived from one nuclear subdivision), and the boundaries between the LA and the BA in humans are more difficult to define given the complex and irregular architecture of the human amygdala.

We validated two additional markers of spatially segregated excitatory neurons. We chose *PEX5L*, as it was the top marker gene of the second excitatory neuron cluster specific to the BA, and *SATB1*, as it was the second highest differentially expressed gene in LA excitatory neurons in both macaques and humans (Figs. 3, A and B, and 4C). In macaques, there was clear spatial segregation of excitatory neurons expressing either *SATB1* or *PEX5L* (fig. S20), with excitatory neurons expressing *SATB1* confined to the LA and neurons expressing *PEX5L* confined to the BA. The density of cells expressing *PEX5L* and *SATB1* was higher in the dorsal, magnocellular portions of the BA and LA.

### Stratification of genetic risk for neuropsychiatric disorders across human amygdalar cell types

**Fig. 8.**
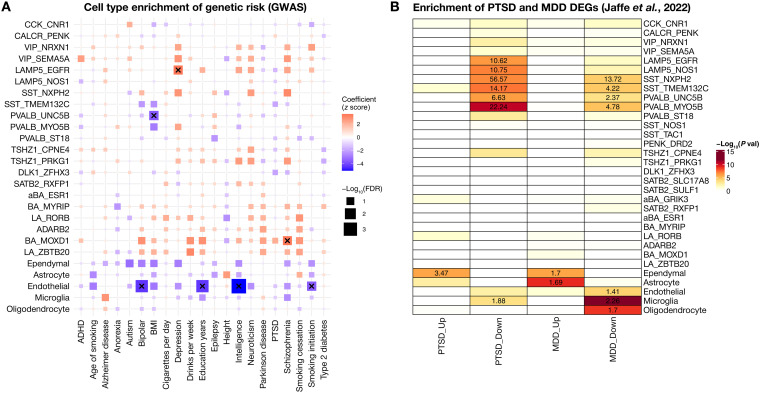
Association of amygdala cell types and subdivisions with psychiatric disorders. (**A**) Heatmap showing LDSC regression coefficient *z* scores for the heritability of different traits (*x* axis) across cell types (*y* axis). “×” denotes FDR-adjusted *P* < 0.05. (**B**) Heatmap displaying the cell type–specific gene set enrichment (overrepresentation) of differentially expressed genes (DEGs) in PTSD and MDD from bulk RNA-seq BLA data by Jaffe *et al.* (*32*). The effect size (odds ratio) is shown for cell types with a *P* value <0.001 (Fisher’s exact test) BMI, body mass index; ADHD, attention-deficit/hyperactivity disorder.

Amygdala dysfunction is a feature of many neuropsychiatric disorders, particularly those associated with fear or anxiety, mood regulation, and addiction (*30*). We took two separate approaches to investigate the potential association of discrete neuronal cell types with neuropsychiatric disorders (Fig. 6). First, to investigate the potential contribution of amygdala cell types to neuropsychiatric risk, we applied stratified-linkage disequilibrium score regression (S-LDSC) (*31*), a method that quantifies the contribution of cell type–specific gene expression to trait heritability using genome-wide association study (GWAS) data (Fig. 8A). Specifically, we used GWAS summary statistics for 21 traits including several neurodegenerative disorders and psychiatric traits [e.g., Alzheimer’s disease, autism, depression, Parkinson’s disease, posttraumatic stress disorder (PTSD), bipolar disorder, and schizophrenia], as well as non–brain-related traits (e.g., height and type 2 diabetes). After correcting for multiple comparisons (FDR *P* < 0.05), we found five cell types with reduced trait heritability and two cell types with increased trait heritability. Specifically, endothelial cells and PVALB_MYO5B neurons contain reduced heritability for bipolar disorder, whereas endothelial cells also show reduced heritability for education years, intelligence, and smoking initiation. In contrast, we found that LAMP5_EGRF inhibitory neurons harbored elevated genetic risk for major depressive disorder (MDD) and excitatory BA_MOXD1 neurons harbored genetic risk for schizophrenia.

To complement our investigation in the heritability of psychiatric risk, we next asked whether cell type–specific gene expression profiles in the amygdala overlap with transcriptional changes observed in individuals with neuropsychiatric disease. We specifically tested whether genes differentially expressed in the BLA of individuals with PTSD or MDD, identified from a large postmortem bulk RNA sequencing (RNA-seq) dataset (*32*), were statistically overrepresented among the marker genes of each cell type (including nonneuronal populations) in our human snRNA-seq dataset. We found that astrocytes and ependymal cells showed the strongest enrichment of MDD–up-regulated genes, while only ependymal cells showed enrichment of PTSD–up-regulated genes (Fisher’s exact test, *P* < 0.001). In contrast, a wide range of GABAergic cell types, including *SST*^+^, *PVALB*^+^, and *LAMP5*^+^ inhibitory neurons, as well as endothelial cells, microglia, and oligodendrocytes showed strong enrichment of genes that were down-regulated in patients diagnosed with either MDD or PTSD (Fisher’s exact test, *P* < 0.001). This is consistent with prior studies implicating immune dysfunction and altered inhibitory signaling in PTSD pathophysiology (*30*, *33*). Together, these complementary approaches highlight a key role for distinct inhibitory neuron classes and nonneuronal cell types in the molecular pathology and genetic risk of neuropsychiatric disorders, reinforcing the amygdala’s central involvement in disease and pointing to specific cellular targets for future mechanistic and therapeutic studies.

**Fig. 9.**
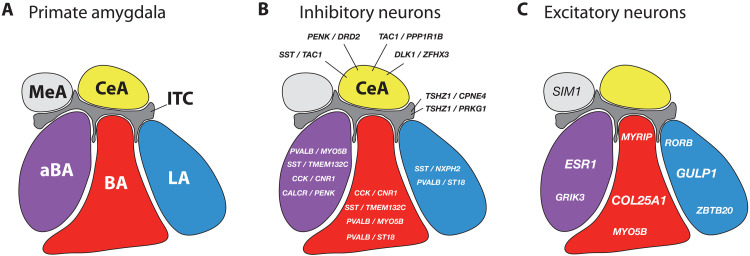
Molecular organization of inhibitory and excitatory neurons across primate amygdala nuclei. (**A**) Summary schematic of primate amygdala subdivisions: MeA (light gray), CeA (yellow), ITCs (dark gray), aBA (purple), BA (red), and LA (blue). (**B**) Highlighted inhibitory neuron types that vary across subdivisions. (**C**) Subdivision-specific excitatory neuron marker genes. Cell types that do not show differential subdivision localization (e.g., *LAMP5*^+^ and *VIP*^+^ inhibitory neurons) are not shown.

In summary, this cross-species atlas reveals that the primate amygdala is highly organized with both inhibitory and excitatory subtypes spatially segregated across major subdivisions (Fig. 9). The finding that psychiatric genetic risk is enriched in specific cell types underscores the relevance of this organization for understanding disease mechanisms. This atlas provides a critical foundation for future efforts to link cell type identity to circuit function, behavior, and disease vulnerability.

## DISCUSSION

Despite neuroanatomical evidence that the amygdala is a hub of interconnected nuclei that mediate specific motivated behaviors across species (*2*), it has remained challenging to understand structure-function relationships in the primate. While there are a number of theories about information flow between subdivisions and interconnected regions (*1*, *16*, *34*–*36*), these have largely gone untested in primates. Contributing to this challenge is a lack of knowledge about the molecular diversity of excitatory and inhibitory neurons within and across individual amygdalar nuclei, which has hindered the ability to capitalize on available knowledge about cytological and histochemical features, as well as connectivity (*5*). Our approach maintained the sensitivity of snRNA-seq and took advantage of the spatial layout of the NHP amygdala to enable targeted sampling of five nuclear subdivisions of the amygdala. This allowed spatially resolved molecular profiling of excitatory and inhibitory neurons, which to date has only been available for rodents. We describe at least 32 different populations of excitatory and inhibitory neurons in the primate amygdala, all of which are highly conserved across human and NHP species and many of which have distinct anatomical origins (Fig. 9). Moreover, we demonstrated the utility of this anatomically defined mesoscale tissue dissection approach by showing how annotations of cell types in the NHP can infer the anatomical origins of human cell types.

We identified 19 distinct types of GABAergic inhibitory neurons that spanned the three major ganglionic eminences and showed substantial variability in their spatial distribution across amygdala subnuclei. In line with previous work (*8*, *14*), we found that the CeA was composed mainly of LGE-derived inhibitory neurons. Among these, we identified two types of *PRKCD^+^* inhibitory neurons that were distinguishable by the expression of *PENK* and *DRD2* versus *TAC1* and *DRD1.* Prior investigations of *PRKCD^+^* inhibitory neurons in rodents focused on D2-mediated effects on aversive learning and fear expression because of its abundant expression in the CeA (*37*). Individual differences in *PRKCD* expression in rhesus macaques are moderately associated with behavioral measures of anxious temperament, and a subset of *PRKCD* inhibitory neurons innervated by SST inhibitory neurons projects to the bed nucleus of the stria terminalis (*11*). Considering the similar developmental origins of the striatum, it is interesting to consider whether differences in D1 and D2 receptor expression among *PRKCD^+^* inhibitory neurons play a similar role in shaping appetitive and aversive responding as in the striatum (*38*). However, D1 and D2 receptor antagonism in the CeA reduces fear learning (*39*) or extinction (*37*). The complexities of dopamine receptor expression within the CeA and *PRKCD^+^* interneuron subtypes warrant further investigation, especially in NHPs.

The ITCs are relatively understudied in primates relative to rodents despite their key role in amygdala function (*12*). Prior neuroanatomical and morphological work in NHPs has respectively confirmed *FOXP2* and *TSHZ1* as conserved markers of ITCs (*40*) and identified at least two distinct spiny and aspiny ITC populations differentiable by the expression of striatum-associated dopamine– and adenosine 3′,5′-monophosphate–regulated phosphoprotein (*PPP1R1B*) (*40*). We identified two distinct clusters of *TSHZ1^+^*/*FOXP2^+^* inhibitory neurons that were highly conserved across NHPs and humans, but neither showed enriched expression of *PPP1R1B* relative to the other. While the TSHZ1_PRKG1 population does contain increased expression of the transcription factor *BCL11B*, which has been shown to be critical to the development of medium spiny projection neurons in the striatum (*41*), further work combining transcriptomic and morphological analyses is needed to delineate these populations. We did, however, find that the *PRKG1*^+^ and *CPNE4*^+^ populations differed in their expression of dopamine D3 receptors (*DRD3*) and serotonin receptors 2C and 7 (*HTR2C* and *HTR7*, respectively; figs. S8 and S13), respectively. Dopamine affects the plasticity of ITC neurons that form synapses with excitatory projection neurons from the LA (*39*), but serotonergic modulation of ITC neuron plasticity and function is understudied. We found that both ITC subtypes were largely sampled from punches targeting the CeA in NHPs; however, we were unable to further spatially localize these cells across the BLA complex. Future experiments can capitalize on our molecular profiling data to use spatial transcriptomics to better map the distribution of these two neuron types in primates.

We additionally found that several MGE- and CGE-derived inhibitory neuron subtypes, including *PVALB*^+^, *SST*^+^, and *CCK*^+^ populations, exhibited notable subdivision-specific distributions across the BLA. Specifically, we found molecularly distinct subpopulations of *SST*^+^ and *PVALB*^+^ across all three major subdivisions of the BLA, while *CCK*^+^ interneurons were enriched in the BA and aBA. This extends prior immunohistochemical studies in monkeys that suggested molecular heterogeneity within major interneuron classes, including *CCK*^+^ neurons distinguished by *CNR1* expression (*10*), *SST*^+^ neurons by *NPY* expression (*42*), and *PVALB*^+^ neurons by *CALB1* (*43*), but were unable to determine clear spatial distinctions. While there have been few functional investigations of these inhibitory populations in NHPs, advances in genetic tools enabling cell type–specific manipulation in rodents have revealed critical roles for *SST*^+^, *PVALB*^+^, and *CCK*^+^ neurons in the regulation of associative plasticity (*44*), control of neural oscillations underlying behavioral states (*45*), and the regulation of fear extinction (*46*). These findings underscore the organizational complexity and regional specialization of inhibitory circuits within the BLA and raise the possibility that distinct subpopulations of interneuron subtypes may contribute differentially to behavior and disease. Mounting evidence implicates the dysfunction of GABAergic signaling in the etiology of psychiatric disorders (*19*, *47*), including addiction, PTSD, and MDD, where reduced SST expression in the human amygdala has been observed in postmortem tissue (*32*, *48*). Our analyses support this, revealing strong enrichment of MDD– and PTSD–down-regulated genes in multiple inhibitory cell types, including *SST*^+^, *PVALB*^+^, and *LAMP5*^+^ neurons. Complementing these transcriptional findings, our stratified heritability analysis identified the selective enrichment of psychiatric risk variants in specific inhibitory and excitatory neuron types, such as LAMP5_EGRF and BA_MOXD1, as harboring elevated genetic risk for depression and schizophrenia, respectively. Together, these results suggest that molecularly and spatially distinct amygdala cell types may differentially contribute to the development of psychiatric disorders.

We additionally identified 13 distinct types of glutamatergic neurons in the primate amygdala that correspond to the pyramidal-like projection neurons identified in prior neuroanatomical studies (*5*), sometimes referred to as principal neurons (*16*). While the differential assignment of functional roles for specific inhibitory neuron subtypes in the amygdala is well documented, heterogeneity among excitatory neurons has not, until recently, been specified beyond their projection anatomy (*2*). Our results strongly argue for the presence of multiple, molecularly specialized glutamatergic neurons in humans, macaques, and baboons and as previously described in the marmoset amygdala (*20*) and rodent amygdala (*18*, *49*, *50*). The observed heterogeneity associates with multiple neurobiological features, one being the nuclear subdivision to which an excitatory neuron cell body localizes. For example, six different types of excitatory neurons were found almost exclusively in either the aBA, BA, or LA. While localization of the *ESR1^+^* neurons in the aBA might have been predicted on the basis of brainwide expression patterns of *ESR1* (*51*, *52*), there is no neuroanatomical precedent for the other nucleus-specific neuronal types we identified.

Prior morphological analyses of amygdala excitatory neurons across species have not convincingly identified more than one pyramidal-like cell type (*5*, *16*). Therefore, cell type–specific differences in the morphology of the glutamatergic cell types we identified are likely quantitative rather than qualitative. The known morphological differences between pyramidal-like excitatory neurons in the amygdala and cortical pyramidal neurons should provide insight into the potential morphological attributes associated with different transcriptomic profiles (*20*, *21*). Among the excitatory neuron cell types identified, we observed the differential expression of a number of transcription factors (*SATB2*, *MEIS1*, *TRPS1*, and *ZBTB20*) implicated in the development of cortical glutamatergic neurons. These transcription factors were specifically enriched in distinct excitatory subtypes, including *SATB2*^+^, SLC17A6_CARTPT, ADARB2, and LA_ZBTB20 populations. Future experiments should use the molecular framework developed by this atlas to identify morphological and connectivity features of pyramidal-like excitatory amygdala neurons, including axonal project targets.

Another potential source of heterogeneity is differences in the sustained excitability of excitatory amygdala neurons. In vitro, pyramidal-like neurons in the amygdala exhibit three types of responses to a prolonged depolarizing input (*16*, *53*, *54*). Using spike frequency adaptation as a quantitative metric, there is a large range in excitability of pyramidal-like neurons in the amygdala. A small minority of neurons responds with a single spike at the stimulus start, which is not accompanied by a sustained hyperpolarization. Most neurons spike repeatedly or in bursts at the start of the stimulus and then exhibit spike frequency adaptation followed by an afterhyperpolarization during which a subsequent response cannot be elicited. The remaining neurons show minimal or no adaptation and fire for the entire stimulus duration. Despite its utility as a quantitative metric, spike frequency adaptation has not been useful in identifying more than two types of excitatory neurons, and it is not correlated with spatial location. When examined in vivo and in response to depolarizing currents, projection neurons in the BA of cats respond with bursts of spikes, whereas projection neurons in the LA respond with slow membrane potential oscillations (*55*). Similar differences are found in the baseline firing rates and excitability of neurons in the BA versus LA during in vivo neural recordings in macaques (*56*). Up-regulated genes in glutamatergic neurons located in the LA versus BA included multiple GABA receptor subunits (*GABRA1* and *GABRB2*) and potassium voltage-gated ion channels (*KCNH5* and *KCBN2*). Neurotransmitter-gated ion channel activity may therefore regulate the postsynaptic membrane potential in LA excitatory neurons. Linking transcriptomic profiles to the physiological characteristics of these neurons will be an important avenue of future research (*57*).

The LA and BA of the primate amygdala differ markedly in terms of their connectivity and function (*5*, *58*, *59*). In comparison to the CeA, the BLA complex has expanded considerably in primates to accommodate the expansion of the prefrontal cortex and visual inputs underlying binocular vision (*60*). Despite many causal experiments in NHPs and humans implicating the amygdala in motivated behaviors (*59*, *61*), a largely unmet goal is direct comparisons of the effects on motivated behaviors after perturbing specific nuclei or cell types (*62*). For example, all prior studies that have manipulated amygdala function in NHPs reported that chemogenetic receptors were detectable in multiple nuclei (*63*–*65*). The identification and spatial validation of *GULP1*, *SATB1*, *COL25A1*, and *PEX5L* as genetic markers that target specific populations of excitatory neurons in NHPs are a critical first step toward using molecular genetic tools to manipulate specific circuits within the primate amygdala. One excitatory neuron subtype localized to the BA, termed BA_MOXD1, showed enriched heritability for schizophrenia. Modulating the activity of these neurons in the BA of nonhuman primates may offer previously unknown opportunities for clinically relevant translational neuroscience.

Prior attempts to annotate neurons from gross dissections of the human amygdala used marker genes identified in rodents (*22*, *23*). Yu and colleagues (*22*) used this approach to identify several types of excitatory neurons that expressed genes that overlapped with the marker genes we identified, including *ESR1*, *COL25A1*, *SATB2*, and *ST8SIA2*, but were unable to confidently assign anatomical origins for each cell type. For example, *ESR1* expression is the highest in the posterior amygdala in rodent atlases, which corresponds to the aBA in the macaque amygdala (*5*). We confirm that *ESR1^+^* excitatory neurons are a highly conserved cell type across NHPs and humans and are localized to the aBA. In addition, we broadly mapped our LA-, BA-, and aBA-enriched cell types to *HGF*^+^, *LAMP5*^+^, and *VGLL3*^+^ cell types that Yu *et al.* (*22*) identified across rodent and primate species. Yu *et al.* (*22*) originally hypothesized that *SATB2^+^* and *LAMP5^+^* excitatory neurons were more prevalent in the LA of the human amygdala. We found that *SATB2* expression was enriched in four specific types of excitatory neurons (*RXFP1^+^*, *SLC17A8^+^*, *SULF1^+^*, *and RORB^+^*) that represented a minority of the excitatory neurons analyzed. In monkeys, these cell types were predominately sampled from the LA or BA. We did note apparent species differences in a few of our SATB2^+^ populations. However, it seems that most of these are likely artifacts because of inconsistent sampling, perhaps of nonamygdalar structures. For example, we found that SATB2_SLC17A8, which was almost exclusively sampled from NHPs in our dataset, showed one-to-one mapping to the Yu *et al.* (*22*) SATB2_ST8SIA2 population in humans but unexpectedly did not show a clear match to any of their macaque cell types. Similarly, our SATB2_RORB population was largely derived from humans but showed moderate mapping to the Yu *et al.* (*22*) DPP10_SATB2 population. This highlights the complexity in acquiring consistent samples across the primate amygdala. SATB2_SULF1 neurons were also primarily sampled from NHPs in our study and did not have a clear match to human cell types in the Yu *et al.* (*22*) dataset. As *SATB2* is an established marker gene of cortical pyramidal neurons, it is possible that these cell types represent the cortical amygdala cell types that were inconsistently sampled across these studies.

Transcriptomic profiling makes it clear that heterogeneity among excitatory and inhibitory amygdala neurons is a rule rather than an exception. While this heterogeneity might eventually be correlated with quantitative differences in neuronal morphology or function, in conjunction with prior neuroanatomical and electrophysiological studies, our data demonstrate that heterogeneity in molecular profiles varies across the amygdaloid complex. Going forward, conceptual and computational models of the amygdala function will need to account for this heterogeneity to accurately predict amygdala-dependent changes in behavior that are relevant for mental health disorders. The identification of conserved genetic markers associated with excitatory neuron populations specific to the BA or LA in NHPs overcomes a major hurdle to building such models. The transcriptomic profiles described here represent a first step to finding genetic elements associated with molecular markers that can be used to build tools for selective manipulation of individual amygdala nuclei in primates. Such studies will be critical to determine the relevance of the nucleus-specific cell types for amygdala-related behaviors and disease.

## MATERIALS AND METHODS

### Postmortem NHP tissue samples

Fresh, unfixed, and flash frozen amygdala tissue punches were acquired from five rhesus macaques and two female olive baboons (table S1). All animals were paired housed on a 12-hour on/12-hour off lighting schedule with ad libitum access to food and water. Animals were fed standard primate chow twice daily and provided fruit and vegetable enrichment daily. Macaques were observed by trained veterinary technicians daily in their home cages. The Institutional Animal Care and Use Committee and the Institutional Biosafety Committee at the ONPRC and Oregon Health and Science University (OHSU) approved all experimental procedures (animal protocol no. 1131_TR01IP00002202), and all of the guidelines specified in the National Institutes of Health Guide for the Care and Use of Laboratory Animals (National Research Council, 2011) were strictly followed.

Necropsies and tissue collections were performed as previously described (*66*, *67*). Animals were sedated with ketamine (10 mg/kg) and then deeply anesthetized with sodium pentobarbital followed by exsanguination. The brain and spinal cord were perfused through the ascending carotid artery with 1 liter of 0.9% ice-cold saline. The brain was then removed from the skull (<30 min postmortem), deposited into an ice-cold bath of saline for transport, and placed into an ice-cold, steel brain matrix (Electron Microscopy Sciences). A custom 3D-printed brain matrix was used to section the baboon brains. Each brain was positioned in the brain matrix with the ventral surface facing up. The anterior medial temporal sulcus, posterior to the temporal pole and anterior to the rhinal sulcus, was identified on the ventral surface of the temporal lobe. A carbon steel knife blade (Thomas Scientific) was inserted into the slot in the brain matrix that was most closely aligned and orthogonal to the beginning of the anterior medial temporal sulcus. Additional knife blades were inserted anterior and posterior to the first knife blade in 2-mm increments. Depending on the size of the brain, this resulted in either two or three brain slabs that encompassed the anterior-to-posterior extent of the amygdala. The resulting brain slabs were then removed from the brain matrix and laid out flat in sterile petri dishes pretreated with RNase-X. The petri dishes rested on an aluminum plate secured to a chamber filled with dry ice. The temperature of the plate was monitored every 5 min with an infrared thermometer to maintain a temperature of −30° to −15°C for up to 2 hours, while tissue punches were acquired and flash frozen.

Brain slabs in which amygdala was clearly visible in each hemisphere, appearing below the anterior commissure or globus pallidus on front and back faces of the slab, were identified, and 1.0- to 2.5-mm-diameter tissue punches were taken through the full width of the slab. For posterior slabs in which the amygdala appeared on the anterior face of the slab and the hippocampus appeared on the posterior face of the slab, tissue punches were collected and carefully removed from the biopsy needle, and the rostral aspect of the punch was retained for nuclear isolation and snRNA-seq. Tissue punches were checked to ensure that they did not contain noticeable quantities of white matter and then inserted into deoxyribonuclease (DNase)– and ribonuclease (RNase)–free 1.5-mm LoBind (Eppendorf Protein LoBind Tube, cat. no. 22431102) microcentrifuge tubes that were inserted into pulverized dry ice. Ethanol was poured onto the dry ice to flash freeze the tissue punches. All tissue punches were acquired less than 90 min postmortem. After acquiring the tissue punches, the brain slabs were postfixed in 4% paraformaldehyde for 48 hours, cryoprotected in 30% sucrose, and then sectioned in 40-μm sections for histological reconstruction of the punch locations.

### Postmortem human tissue samples

Postmortem human brain tissue was obtained at the time of autopsy with informed consent from the legal next of kin through the Office of the Chief Medical Examiner of the State of Maryland, under the Maryland Department of Health IRB (Institutional Review Board) protocol no. 12-24, the Departments of Pathology at Western Michigan University Homer Stryker MD School of Medicine, and the University of North Dakota School of Medicine and Health Sciences, and through Gift of Life Michigan, all under WCG IRB protocol no. 20111080. Demographics for the five donors are listed in table S2. Details of tissue acquisition, handling, processing, dissection, clinical characterization, diagnoses, neuropathological examinations, and quality control measures have been described previously (*68*). Frozen coronal brain slabs containing the amygdala at the level of clearly visible caudate nucleus and putamen (separated by the internal capsule), globus pallidus external and internal segments, fornix, and the anterior commissure were selected for dissections. Tissue blocks (~10 mm by 20 mm) containing the amygdala were dissected under visual guidance with a handheld dental drill. Blocks were stored in sealed cryogenic bags at −80°C until cryosectioning and between tissue collection rounds. At the time of cryosectioning, tissue blocks were acclimated to the cryostat (Leica CM3050) at −14°C and mounted onto chucks, ~50-μm tissue was trimmed from the block to achieve a flat surface, and several 10-μm sections were collected for quality control [RNAscope and hematoxylin and eosin (H&E) staining]. After identification of the boundaries of the BLA complex, blocks were again acclimated to the cryostat, mounted onto chucks, and scored with a razor to isolate the BLA. One hundred–micrometer sections of the BLA were collected in prechilled 2-ml DNase- and RNase-free microcentrifuge tubes (Eppendorf Protein LoBind Tube, cat. no. 22431102) for a total of 70 to 100 mg of tissue and stored at −80°C until nuclear isolation.

### Anatomical validations and quality control of human tissue blocks (H&E and RNAscope)

Before collecting sections for snRNA-seq experiments, tissue blocks were cut and 10-μm sections were collected to complete two quality control steps: (i) H&E staining to assess the gross neuroanatomical structure of the block and (ii) smFISH (fluorescence multiplex RNAscope). H&E staining and RNAscope were performed according to the manufacturer’s instructions, and images were acquired using an Aperio CS2 slide scanner (Leica) or a NikonAXR (Nikon Instruments). For RNAscope, probes for established marker genes (ACD Bio) were used to identify white matter and subnuclei of the amygdala, including *MBP* (cat. no. 411051), *SLC17A7* (cat. no. 415611), *COL25A1* (cat. no. 1187021), *GULP1* (cat. no.1095761), *PEX5L* (cat. no. 1185381), and *SATB1* (cat. no. 454621).

### RNAscope smFISH

Fresh frozen amygdalae from the rhesus macaque donor and human donor were sectioned at 10 μm and stored at −80°C. In situ hybridization assay was conducted using RNAscope Multiplex Fluorescent Reagent Kit v2 (cat. no. 323100, ACD, Hayward, CA) following the manufacturer’s protocol. In summary, the tissue sections were fixed in 10% neutral buffered formalin solution (cat. no. HT501128-4L, Sigma-Aldrich, St. Louis, MO) for 30 min at room temperature. Sections were dehydrated with serial ethanol washes, pretreated with hydrogen peroxide for 10 min at room temperature, and treated with protease IV for 30 min. Four different probe combinations were used at one time to identify marker gene expression in the BLA with the following probes: Hs-SLC17A7 (cat. no. 415611-C4, ACD, Hayward, CA), Hs-COL52A1 (cat. no. 1187021-C2, ACD, Hayward, CA), Hs-GULP1 (cat. no. 1095761-C3, ACD, Hayward, CA), Hs-MBP (cat. no. 411051-C4, ACD, Hayward, CA), Hs-PEX5L (cat. no. 1185381-C2, ACD, Hayward, CA), Hs-SATB1 (cat. no. 454621, ACD, Hayward, CA), Mmu-GULP1-C3 (cat. no. 1568911-C3, ACD, Hayward, CA), and Mmu-MBP-C4 (cat. no. 1006431-C4, ACD, Hayward, CA). After labeling the probes, sections were stored overnight in 4× SSC buffer. Following amplification steps (AMP1 to AMP3), the probes were fluorescently labeled using opal dyes (PerkinElmer, Waltham, MA; diluted 1:500) and counterstained with DAPI (4′,6-diamidino-2-phenylindole) to mark the nucleus. Sections were imaged using 2× to 40× objectives on NikonAXR (Nikon Instruments).

### NHP nuclear isolation and snRNA-seq

Flash-frozen tissue punches collected from the different subdivisions of the amygdala of macaques (four subdivisions) and baboons (two subdivisions) were dissociated into individual nuclei following the manufacturer’s instructions (protocol CG000366, 10x Genomics). Briefly, a chilled lysis buffer was added to a chilled glass dounce, and the cryosections were transferred while frozen. Sections were homogenized using 5 to 15 strokes with both loose and tight-fit prechilled pestles. Then, the lysis buffer was neutralized with a resuspension buffer. The homogenate was strained through a 70-μm cell strainer, followed by a 40-μm cell strainer. The nucleus suspension was centrifuged in a 29 to 50% OptiPrep gradient (Sigma-Aldrich) at 3000*g* for 20 min at 4°C to separate intact nuclei from debris. The pelleted nuclei was resuspended and centrifuged at 500*g* at 4°C for 5 min, and the supernatant was removed for a total of three times. Nuclei were inspected in a counting chamber for intact, bright, nongranular cell morphologies, indicating high viability and successful debris removal. Approximately 10,000 single nuclei were captured for each sample in a single channel on the 10× Chromium controller, and snRNA-seq libraries (Chromium Next GEM Single Cell 3′ kit version 3.1) were generated following the manufacturer’s instructions. Libraries were sequenced to an average depth of 20,000 read pairs per nucleus in a NovaSeq 6000 at Oregon Health and Science University Massively Parallel Sequencing Shared Resource according to 10x Genomics’ specifications. All samples were processed and sequenced at the same time to avoid batch effects. For the baboon samples, nuclei were extracted from flash-frozen tissue and enriched for intact, single nuclei from neurons using fluorescence-activated nuclear sorting (FANS) based on NeuN immunolabeling (using Rabbit anti-NeuN monoclonal antibody directly conjugated to Alex Fluor 594; Cell Signaling Technology, cat. no. 90171S) and DAPI staining using a slightly modified version of a previously published protocol (*69*). FANS parameters including NeuN gating thresholds and nuclear recovery rates were established for each experiment using unstained control nuclei, and NeuN+/DAPI+ nuclei were isolated with FANS (BD Influx) such that ~20,000 nuclei were used as input into each Next GEM reaction. Library generation and sequencing was as described for macaques.

### Human nuclear isolation and snRNA-seq

Using 100-μm cryosections collected from each donor, we conducted snRNA-seq using 10x Genomics Chromium Single Cell Gene Expression 3′ kit version 3.1. Approximately 70 to 100 mg of tissue was collected from each donor, placed in a prechilled 2-ml microcentrifuge tube (Eppendorf Protein LoBind Tube, cat. no. 22431102), and stored at −80°C until the time of experiment. Nucleus preparations were conducted according to the 10x Genomics customer-developed “Frankenstein” nuclear isolation protocol, as previously described in (*23*), with modifications designed to optimize the protocol for use with cryosections. Briefly, chilled EZ lysis buffer (MilliporeSigma, no. NUC101) was added to the LoBind microcentrifuge tube containing cryosections, the tissue was fragmented by pipette mixing, this lysate was transferred to a chilled glass dounce, and the tube was rinsed with additional EZ lysis buffer, which was added to the respective dounce. Sections were homogenized using 10 to 20 strokes with both loose and tight-fit prechilled pestles, and the homogenate was strained through a 70-μm cell strainer. After lysis, samples were centrifuged at 500*g* at 4°C for 5 min, the supernatant was removed, and the pellet was resuspended in EZ lysis buffer and recentrifuged. The supernatant was removed, and wash/resuspension buffer [phosphate-buffered saline containing 0.5% bovine serum albumin (Jackson ImmunoResearch, no. 001-000-162)] was added to the pellet. Upon resuspension, the samples were spun again, and this wash process with wash/resuspension buffer was completed three times. Nuclei were labeled with Alexa Fluor 488–conjugated anti-NeuN (MilliporeSigma, cat. no. MAB377X) diluted 1:1000 in a nuclear stain buffer [1× phosphate-buffered saline, 3% bovine serum albumin, and RNase inhibitor (0.2 U/μl)] by incubating at 4°C with continuous rotation for 1 hour. Proceeding NeuN labeling, nuclei were washed once in stain buffer, centrifuged, and resuspended in wash/resuspension buffer. Nuclei were labeled with propidium iodide (PI) at 1:500 in wash/resuspension buffer and subsequently filtered through a 35-μm cell strainer. FANS was performed using a Bio-Rad S3e Cell Sorter at the Lieber Institute for Brain Development. Gating criteria were selected for whole, singlet nuclei (by forward/side scatter), G_0_/G_1_ nuclei (by PI fluorescence), and neuronal nuclei (by Alexa Fluor 488 fluorescence). Nine thousand nuclei were sorted into a tube on the basis of PI+ and NeuN+ fluorescence to facilitate the enrichment of neurons, which resulted in 81.9% neurons across all samples. Samples were collected over two rounds, each including two or three donors. All samples were sorted into reverse transcription reagent master mix from the 10x Genomics Single Cell 3′ Reagents kit (without an enzyme). A reverse transcription enzyme and water were added to bring the reaction to full volume after sorting. cDNA synthesis and subsequent library generation were performed according to the manufacturer’s instructions for the Chromium Next GEM Single Cell 3′ version 3.1 (dual-index) kit (CG000315, revision E, 10x Genomics). Samples were sequenced on a Nova-seq 6000 (Illumina) at the Johns Hopkins University Single Cell and Transcriptomics Sequencing Core.

### Processing of raw snRNA-seq data

All FASTQ files for snRNA-seq libraries were aligned using 10x Genomics software, cellranger count (version 7.0.0). Libraries from the human donors were aligned to the human genome reference (GRCh38/Hg38, Ensembl release 98), rhesus macaque samples were aligned to the macaque (*Macaca mulatta*) genome reference (Mmul_10, Ensembl release 110), and baboon samples were aligned to the olive baboon (*Papio anubis*) genome reference (Panubis1.0, National Center for Biotechnology Information release 104). Feature-barcode files were analyzed in R version 4.3.1 within the Bioconductor framework (version 3.17), unless otherwise stated. Empty droplets were identified and removed using the emptyDrops function from the DropletUtils package using a data-driven threshold. To identify droplets containing more than one nucleus (i.e., doublets), sample-specific doublet scores were calculated using computeDoubletDensity from snDblFinder with the top 2000 highly variable genes. Droplets with a score greater than or equal to 2.75 were excluded from downstream analyses.

For human and baboon samples, quality control was performed by computing sample-wise median absolute deviation (MAD) thresholds for the total number of UMIs, the number of unique detected genes, and the proportion of reads mapping to mitochondria. Individual cells were considered low quality if they exceeded less than −3 MADs for the total UMI and unique genes and/or greater than +3 MADs for mitochondrial ratio per sample. The total UMI and unique gene quality control metrics for macaque samples displayed bimodal distributions because of the inclusion of both neuronal and nonneuronal cell types, resulting in no nuclei being excluded using the 3 MAD thresholds. For this reason, minimum thresholds of 600 UMI and 500 unique genes were used instead. Additionally low-quality clusters were dropped. These preprocessing steps resulted in a final total of 15,132 human, 82,524 macaque, and 34,156 baboon nuclei.

To allow for cross-species comparisons, genes were subset to include only one-to-one orthologs using the convert_orthologs function from the orthogene package using the gprofiler method. For NHP data, input_species was set to either “macaque” or “baboon” and the output_species variable was set to “human,” and the strategy for handling non–one-to-one matches was set to “drop_both_species.” The intersection was then taken for the one-to-one orthologs between the three species. After filtering out mitochondrial and lowly expressed genes, this resulted in a total of 13,842 common genes across human and NHP species.

### Feature selection, cross-species integration, and fine-resolution clustering

Feature section, dimensionality reduction, cross-species integration, and clustering were all carried out using Seurat v4 workflows. The combined data were first split by subjects and species. All data underwent normalization and variance stabilization using the SCTransform function while regressing out mitochondrial percentage, the top 2000 highly variable genes were selected using the FindVariableFeatures function, and the data were centered and scaled using the ScaleData function. Features used for integration were then selected using the SelectIntegrationFeatures function. These features were then centered and scaled for each data split before performing principal components analysis (PCA). Data integration across subjects was performed using the IntegrateData function using anchors determined using FindIntegrationAnchors. Identical procedures were used for within-species batch correction and clustering. Default parameters were used for all functions.

After cross-species integration, data were rescaled and PCA was run on the integrated data. Data were then clustered (FindClusters function) on the top 30 principal components using the Leiden algorithm and a resolution of 0.5. Data were visualized with the UMAP dimensionality reduction technique via Seurat’s RunUMAP function with the top 30 principal components. Default parameters were used for all functions, unless otherwise specified. These initial clusters were then grouped on the basis of canonical marker gene expression into either excitatory neurons (*SNAP25*^+^, *SLC17A7*^+^, and *SLC17A6*^+^), inhibitory neurons (*SNAP25*^+^, *GAD1*^+^, and *GAD2*^+^), or nonneuronal cell types (*SNAP25*^−^). Inhibitory neurons were also identified on the basis of canonical marker genes such as *SST*, *PVALB*, *CARTPT*, *VIP*, *CCK*, *LAMP5*, *PRKCD*, *TSHZ1*, *NTS*, and *PENK.* To obtain fine-resolution cell type clusters, both excitatory and inhibitory neuron types were subclustered by subsetting the data to exclusively excitatory or inhibitory neurons before rerunning the above Seurat integration and cluster workflows. Subcluster quality was assessed with the pairwise modularity ratio using the pairwiseModularity function from the bluster package, which determined how separated each cluster is from one another. Any clusters that showed poor separation (i.e., low modularity score) were collapsed to a single cluster.

### Cell type–specific marker genes, annotations, and visualizations

For excitatory neurons, fine-resolution cell type annotations were performed automatically using cluster-specific marker genes detected by the findMarkers_1vAll function from the DeconvoBuddies package using the log-normalized counts with ~species used as a blocking factor. Annotation names were then assigned on the basis of the top two marker genes for each cell type as *Gene1_Gene2*. The same method was used for annotating inhibitory neurons, except that canonical marker genes based on established classes of GABAergic cell types (*PVALB*, *SST*, *VIP*, *CCK*, *PRKCD*, *NTS*, *CARTPT*, *LAMP5*, and *TSHZ1*) were listed in place of *Gene1*. Nonneuronal cell types were also broadly labeled on the basis of canonical gene expression for astrocytes (*GFAP* and *SLC1A1*), OPCs (*CD9*), oligodendrocytes (*MBP*), ependyma (*FOXJ1*), endothelial (*RGS5*), and microglia (*TMEM119*).

Heatmaps visualizing marker gene expression for excitatory, inhibitory, and nonneuronal cell types were all generated using the ComplexHeatmap package. For this, the mean log-normalized counts were aggregated across cells using the aggregateAcrossCells function from the scuttle package. Species proportions were calculated as the proportion of nuclei sampled from each species within each fine cell type. Because precise punches of all regions (LA, BA, aBA, and CeA) were only collected within macaques, the subdivision proportions were calculated as the proportion of nuclei sampled from each subdivision only within the macaque dataset.

### Cross-species comparisons of fine cell types using MetaNeighbor

We used the MetaNeighbor package to assess cell type conservation across species using the unsupervised approach. This method evaluates the similarity between cell types across different datasets (species) by using one cell type as a training set and another as a test set, averaging the AUROC scores across these comparisons to create a matrix that reflects the degree of similarity between cell types across species. An AUROC score of 0.5 indicates random classification, whereas higher scores indicate high accuracy in predicting cell types across species. This allows us to determine the degree of conservation across cell types. MetaNeighbor has been shown to be robust to class imbalances. Highly variable genes (selected with MetaNeighbor’s variableGenes function) were used as input features to the MetaNeighborUS function with the parameters study_id = “species,” cell_type = “fine_celltype,” fast_version = TRUE, one_vs_best = TRUE, and symmettric_output = FALSE. Heatmaps and box plots were generated from the resulting AUROC matrix using the ComplexHeatmap and ggplot packages, respectively.

### Comparisons to existing datasets

To evaluate the concordance of cell type–specific transcriptional signatures across datasets and species, we used the registration_wrapper and layer_stat_cor functions from the spatialLIBD R package (*70*). For each dataset, we first computed differential expression t-statistics for each cell type relative to all other cell types using the registration model framework implemented in spatialLIBD. This was performed separately for each dataset by providing the relevant cell type labels and sample identifiers to the registration_wrapper function.

To quantify transcriptional similarity across datasets, we extracted the resulting t-statistics for each cell type and computed Pearson correlations using the layer_stat_cor function. Correlations were calculated on the basis of the top 100 marker genes per cell type. The resulting correlation matrices summarize the similarity of cell type–specific gene expression patterns between datasets or species. Correlation plots were generated using the layer_stat_cor_plot function.

### Within-species cell type clustering and label transfer

To confirm that cross-species integration was not masking species-specific cell types, broad and fine cell types were identified within the macaque dataset as described above. After fine cell type annotations were obtained for both inhibitory and excitatory cell types, datasets for each species underwent normalization and variance stabilization using the SCTransform function while regressing out mitochondrial percentage and then underwent PCA dimensionality reduction. Labels were transferred from the macaque dataset to human and baboon datasets using a reference-to-query method via Seurat’s FindTransferAnchors and TransferData functions using the top 30 principal components. The median prediction accuracy per cell type was used to assess the strength of cross-species label transfer.

### Compositional analysis of cell type abundances using linear mixed modeling

All compositional data analyses were carried out using a combination of the variancePartition, dreamlet, and crumblr R/Bioconductor packages (*26*). In short, we aggregated fine cell type counts from macaques to the sample level using aggregateToPseudoBulk from dreamlet. The crumblr function from the crumblr package was then used to compute the CLR transformation to ensure data normality before linear modeling. Differential abundance testing was then conducted using the dream function from the variancePartition package, fitting a precision-weighted linear mixed model to the cell type proportions estimated from the crumblr object (*29*). Various models were implemented to test specific hypotheses, as described below.

### Differential abundance testing of inhibitory neurons in the CeA versus BLA

Given the unique developmental origins and molecular specialization of inhibitory neurons in the CeA, such as the presence of LGE-derived subtypes not found in the basolateral complex, we first performed a targeted differential abundance test comparing inhibitory neuron subtypes in the CeA versus BLA. The BLA was defined by collapsing the LA, BA, and aBA subdivisions, while the CeA was retained as a separate group. Differential abundance modeling was performed using dream with the following linear mixed model

$$Y_{\mathrm{ij}}=\beta_{{\text{Region}}_{\mathrm{ij}}}+\beta_{{\text{Sex}}_{\mathrm{ij}}}+\beta_{{\text{Species}}_{\mathrm{ij}}}+U_{j}+Z_{\mathrm{ij}}$$ (1)

where $Y_{\mathrm{ij}}$ is the CLR-transformed proportion of inhibitory neuron type i in subject j , $\beta_{{\text{Region}}_{\mathrm{ij}}}$ is the fixed effect of region (i.e., CeA versus BLA), $\beta_{{\text{Sex}}_{\mathrm{ij}}}$ is the fixed effect of sex, $\beta_{{\text{Species}}_{\mathrm{ij}}}$ is the fixed effect of species, $U_{j}$ represents the random effect of intersubject variability, and $Z_{\mathrm{ij}}$ is the residual error. Significantly enriched or depleted cell types in the CeA versus BLA were identified using empirical Bayes moderation eBayes to stabilize variance estimates and improve statistical power, and multivariate testing was performed using treeTest on a cluster tree generated by buildClusterTreeFromPB.

### Differential abundance testing of inhibitory neurons across BLA subdivisions

To assess the distribution of inhibitory neuron subtypes across subdivisions of the BLA, we performed compositional analysis using crumblr. Because dorsal tissue punches of the BLA may include cells from the adjacent CeA, we first excluded all CeA samples and removed cell types known to be CeA-specific (DLK1_ZFHX3, PENK_DRD2, TAC1_PPP1R1B, and SST_TAC1). The remaining data included inhibitory neurons from the LA, BA, and aBA. For this, a similar workflow and linear model was used as above

$$Y_{\mathrm{ij}}=0+\beta_{\text{Subregio}n_{\mathrm{ij}}}+\beta_{\text{Se}x_{\mathrm{ij}}}+\beta_{\text{Specie}s_{\mathrm{ij}}}+U_{j}+Z_{\mathrm{ij}}$$ (2)

where $Y_{\mathrm{ij}}$ is the CLR-transformed proportion of inhibitory neuron type i in subject j , $\beta_{\text{Subregio}n_{\mathrm{ij}}}$ is the fixed effect of BLA subdivisions, $\beta_{\text{Se}x_{\mathrm{ij}}}$ is the fixed effect of sex, $\beta_{\text{Specie}s_{\mathrm{ij}}}$ is the fixed effect of species, $U_{j}$ represents the random effect of intersubject variability, and $Z_{\mathrm{ij}}$ is the residual error.

To formally test pairwise subdivision differences, we used the makeContrastsDream function to define the following linear contrasts:

BA versus LA

$$H_{0}:\beta_{\text{BA}}-\beta_{\text{LA}}=0$$ (3)

aBA versus LA

$$H_{0}:\beta_{\text{aBA}}-\beta_{\text{LA}}=0$$ (4)

aBA versus BA

$$H_{0}:\beta_{\text{aBA}}-\beta_{\text{BA}}=0$$ (5)

Models were again fit using dream, followed by empirical Bayes shrinkage with the eBayes function to stabilize variance estimates and improve statistical power.

### Differential abundance testing of excitatory neurons across subdivisions

We next fit a linear mixed model to determine cell type composition differences of excitatory neurons across the amygdala. The same model and contrasts used for comparing inhibitory neurons across BLA subdivisions (Eq. 2) were used here, except that it contained all excitatory neuron types across all samples, including the CeA.

The CeA of the amygdala neighbors the medial nucleus of the amygdala, which is known to contain distinct populations of vGlut2-expressing neurons. To determine whether we captured any of these medial nucleus cell types, we defined an additional linear contrast to test the difference in excitatory neurons derived from CeA samples compared to the average composition of the basolateral complex:

CeA versus BLA complex (LA + BA + aBA) average

$$H_{0}:\beta_{\text{CeA}}-\frac{1}{3}\left(\beta_{\text{LA}}+\beta_{\text{BA}}+\beta_{\text{aBA}}\right)=0$$ (6)

### Differential abundance testing across the dorsoventral axis within the LA and BA

In macaque LA and BA samples, punches were taken at varying positions along the dorsoventral (DV) axis, and metadata regarding the DV axis location were retained. To test for potential differences in excitatory neuron composition across the DV axis within LA and BA subdivisions, we subset the macaque dataset to only dorsal or ventral samples from the LA and BA and modeled an interaction between subdivision and the DV axis. The following linear mixed model was used

$$Y_{\mathrm{ij}}=\beta_{\text{Subregio}n_{\mathrm{ij}}}+\beta_{\text{Se}x_{\mathrm{ij}}}+\beta_{DV_{\mathrm{ij}}}+\beta_{\text{DV}\times \text{Subregio}n_{\mathrm{ij}}}+U_{j}+Z_{\mathrm{ij}}$$ (7)

where $Y_{\mathrm{ij}}$ is the CLR-transformed proportion of inhibitory neuron type i in subject i , $\beta_{\text{Subregio}n_{\mathrm{ij}}}$ is the fixed effect of BLA subdivisions, $\beta_{\text{Se}x_{\mathrm{ij}}}$ is the fixed effect of sex, $\beta_{DV_{\mathrm{ij}}}$ is the fixed effect of the DV axis, $\beta_{\text{DV}\times \text{Subregio}n_{\mathrm{ij}}}$ is the interaction of the DV axis within BLA subdivisions, $U_{j}$ represents the random effect of intersubject variability, and $Z_{\mathrm{ij}}$ is the residual error.

### Cell type tree testing in compositional analyses

We used treeTest() to perform multivariate testing over a dendrogram of cell types, generated using buildClusterTreeFromPB(), allowing for structured borrowing of information across related clusters. This provided effect size estimates and FDRs for each cell type branch.

### Putative subdivision superclusters

Excitatory cell type clusters found to be enriched in LA (*LA_ZBTB20* and *LA_RORB*), BA (*BA_MOXD1* and *BA_MYRIP*), or aBA (*aBA_ESR1* and *aBA_GRIK3*) samples from NHPs were classified as putative LA and BA excitatory clusters in all species. These classifications were used in subsequent analyses for comparisons across amygdala subdivisions.

### Pseudobulk differential expression analysis

Novel marker genes for amygdala subdivision were identified using pseudobulk differential expression analysis. The mRNA counts of excitatory neurons from independent LA (*n* = 15; 12 macaques and 3 baboons) and BA (*n* = 17; 13 macaques and 4 baboons) samples were aggregated (i.e., pseudobulked) using the aggregateAcrossCells function from the scuttle package. Lowly expressed genes were filtered out using the filterByExpr function from the edgeR package with the group variable set to subdivision.

Pseudobulk DE analysis was carried out using Dream (*29*) from the variancePartition package to account for repeated measures across subjects by using random effects to model intersubject variability. Count data were aggregated across cells by subdivision, species, sample, and subject using the aggregateAcrossCells function from the scuttle package, yielding pseudobulk expression profiles for each biological replicate. Gene-level counts were then normalized using the trimmed mean of M-values method via the calcNormFactors function from the edgeR package, and the linear mixed model was specified as

$$Y_{\mathrm{ij}}=\beta_{\text{Subregio}n_{\mathrm{ij}}}+\beta_{\text{Se}x_{\mathrm{ij}}}+\beta_{\text{Specie}s_{\mathrm{ij}}}+U_{j}+Z_{\mathrm{ij}}$$ (8)

where $Y_{\mathrm{ij}}$ is the expression of gene i in subject j , $\beta_{\text{Subregio}n_{\mathrm{ij}}}$ is the fixed effect of BLA subdivisions, $\beta_{\text{Se}x_{\mathrm{ij}}}$ is the fixed effect of sex, $\beta_{\text{Specie}s_{\mathrm{ij}}}$ is the fixed effect of species, $U_{j}$ represents the random effect of intersubject variability, and $Z_{\mathrm{ij}}$ is the residual error. The voomWithDreamWeights function was used to estimate observation-level precision weights, followed by model fitting with dream and empirical Bayes moderation using eBayes. Differential expression statistics were extracted using topTable, focusing on the contrast between LA and BA subdivisions. To visualize cross-species expression patterns of putative subdivision-enriched genes, we aggregated data by subdivision supercluster (see above), species, and sample and computed counts per million (CPM) using edgeR’s cpm function.

### Correlation of marker gene t-statistics to find conserved marker genes

To determine the cross-species conservation of LA and BA marker genes, we ran a correlation analysis of the standard logFC of marker genes associated with amygdala subdivisions. Excitatory neurons were first subset to just include nuclei within the putative LA, BA, and aBA cell type clusters. Marker genes for the putative subdivisions were then found within each species using the findMarkers_1vAll function from the DeconvoBuddies package while including Sample as a blocking factor. We then calculated the Pearson correlation coefficient (cor function with default parameters from base R) to compare the subdivision marker gene logFC between species.

### Gene set enrichment analysis of psychiatric disorder risk genes

To assess the cell type–specific enrichment of psychiatric disorder risk genes, we performed gene set enrichment analysis using bulk RNA-seq differential expression results from the human BLA in PTSD and MDD (*32*). Genes were filtered at *P* < 0.01 for PTSD and MDD and categorized into up-regulated and down-regulated sets on the basis of logFC direction. Using our human amygdala dataset as a cell type reference, pseudobulk differential expression modeling was performed with the registration_wrapper function from the spatialLIBD package, which computes gene-level t-statistics and FDRs for each annotated cell type. Enrichment was performed using a custom Fisher’s exact test framework via the gene_set_enrichment function, comparing each gene set (PTSD-up, PTSD-down, MDD-up, and MDD-down) against genes significantly enriched in each cell type (FDR < 0.1). Odds ratio and *P* values were computed for each gene set-cell type pairing. Results were then visualized with the gene_set_enrichment_plot function, displaying odds ratios across cell types ordered by fine cell type annotation.

### Stratified linkage disequilibrium score regression (S-LDSC)

Before S-LDSC, we defined gene sets for each set of fine cell type classes using human data. In short, fine cell types were first filtered to exclude human cell types with less than 70 total cells before being pseudobulked using the aggregateAcrossCells function using default parameters. Genes were then filtered to retain only protein-coding genes that were expressed above background levels in a sufficient number of cells using the filterByExpr function from the edgeR package. This resulted in a total of 15,128 genes. The resulting pseudobulked dataset was then normalized by first calculating normalization factors (calcNormFactors function) and then normalizing to counts per million (cpm function) using the edgeR package.

Genes sets for use in S-LDSC were created by calculating the top 10% most highly expressed genes within each cell type. To further normalize gene expression, the expression of each gene was divided by the gene’s total expression across all cell types such that each gene’s expression values summed to 1. We then calculated the 90th percentile threshold for each cell type to define the top 10% highly expressed genes per cell type. We then added a 100-kb window upstream and downstream of the transcribed region of each gene in the gene sets to construct a genome annotation for each unique cell type label.

We conducted S-LDSC to assess the heritability of brain-related traits across the gene sets associated with each human cell type. To determine whether the observed enrichments were specific to brain-related traits, we included human height and type 2 diabetes as negative control traits. GWAS summary statistics for all 21 traits were obtained from the respective studies (*71*–*79*). We performed S-LDSC using baseline LD model version 2.2, which includes 97 annotations to account for linkage disequilibrium with other functional genomic annotations, per the guidelines from the LDSC resource website (https://alkesgroup.broadinstitute.org/LDSCORE). We used single-nucleotide polymorphisms (SNPs) from HapMap Project Phase 3 as the regression SNPs and SNPs from the 1000 Genomes Project (European ancestry samples) as the reference SNPs, both sourced from the LDSC resource. To assess the specific contribution of each gene set to trait heritability, we relied on the *z* score of per-SNP heritability provided by S-LDSC. This *z* score allows us to isolate the unique impact of each gene set while considering the influence of other annotations in the baseline model. *P* values were calculated from the *z* scores under the assumption of a normal distribution, and we applied the Benjamini and Hochberg procedure to control the FDR.
