# Supplementary material for: Transcriptomic diversity of amygdalar subdivisions across humans and nonhuman primates
Source: Sci Adv. 2025 Sep 17;11(38):eadw1029. doi: 10.1126/sciadv.adw1029 (PMC12442850; doi:10.1126/sciadv.adw1029)
Supplement: Supplementary file 1 — Figs. S1 to S20 Tables S1 to S3 [file sciadv.adw1029_sm.pdf]

Science Advances
AAAS

Supplementary Materials for

Transcriptomic diversity of amygdalar subdivisions across humans and nonhuman primates

Michael S. Totty *et al.*

Corresponding author: Vincent D. Costa, vincent.d.costa@emory.edu; Stephanie C. Hicks, shicks19@jhu.edu; Keri Martinowich, keri.martinowich@libd.org

Sci. Adv. **11**, eadw1029 (2025)
DOI: 10.1126/sciadv.adw1029

This PDF file includes:

Figs. S1 to S20
Tables S1 to S3

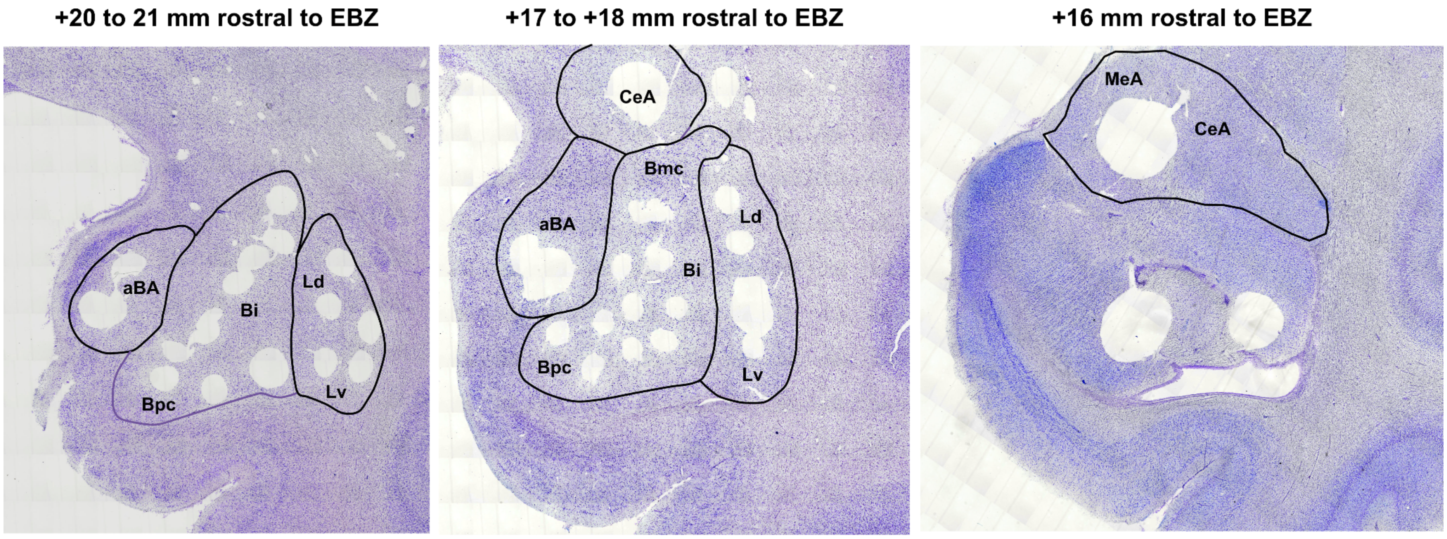

Supplemental Figure 1. Histological reconstructions of representative tissue punches illustrating the targeted dissection strategy. Tissue punches were taken from fresh, unfrozen tissue using gross neuroanatomical landmarks, such as the white matter tracts segregating the different nuclear subdivisions. Nissl staining after the tissue was fixed was performed to confirm accurate targeting of distinct nuclear subdivisions. Representative sections are shown from 3 different macaques, representing the rostral to caudal extent of the sampling of all four major subdivisions. Additional tissue punches from the hippocampus were taken but not sequenced. Tissue perforations that are not circled represent vasculature or tears that occurred during tissue processing. aBA, accessory basal nucleus; CeA, central nucleus; Bmc, magnocellular division of the basal nucleus, Bi, intermediate division of the basal nucleus; Bpc, parvocellular division of the basal nucleus; Ld, dorsal division of the lateral nucleus; Lv, ventral division of the lateral nucleus; MeA, medial nucleus; EBZ, ear bar zero.

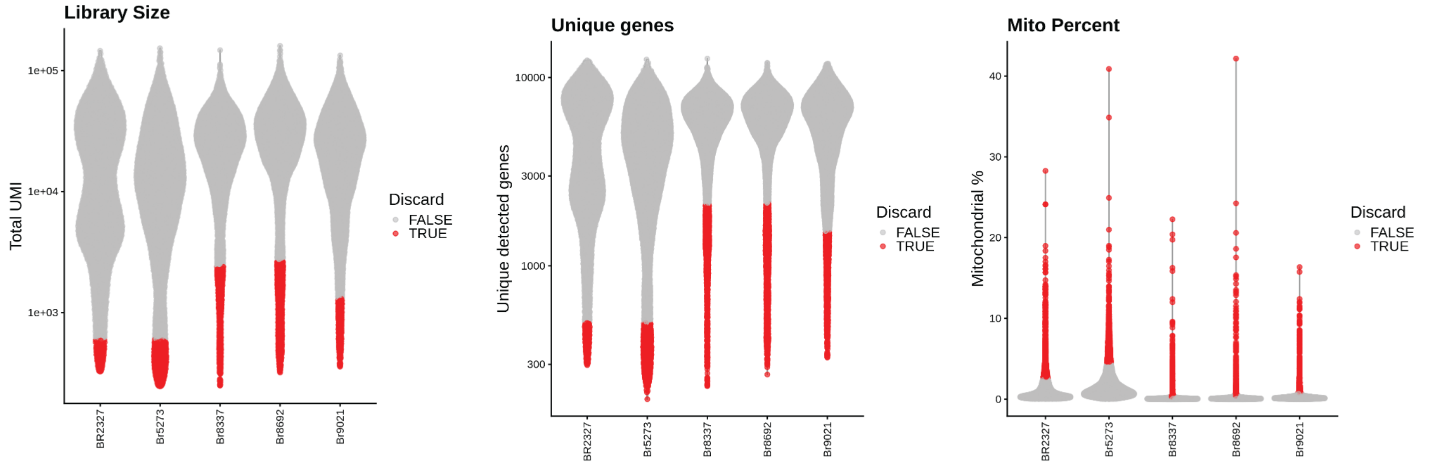

Supplementary Figure 2: Quality control metrics for human samples. Violin plots displaying library size, number of unique genes, and mitochondrial percent metrics across the five human samples (n=5), each from a unique donor. Red data points were considered low-quality nuclei and were excluded from downstream analyses.

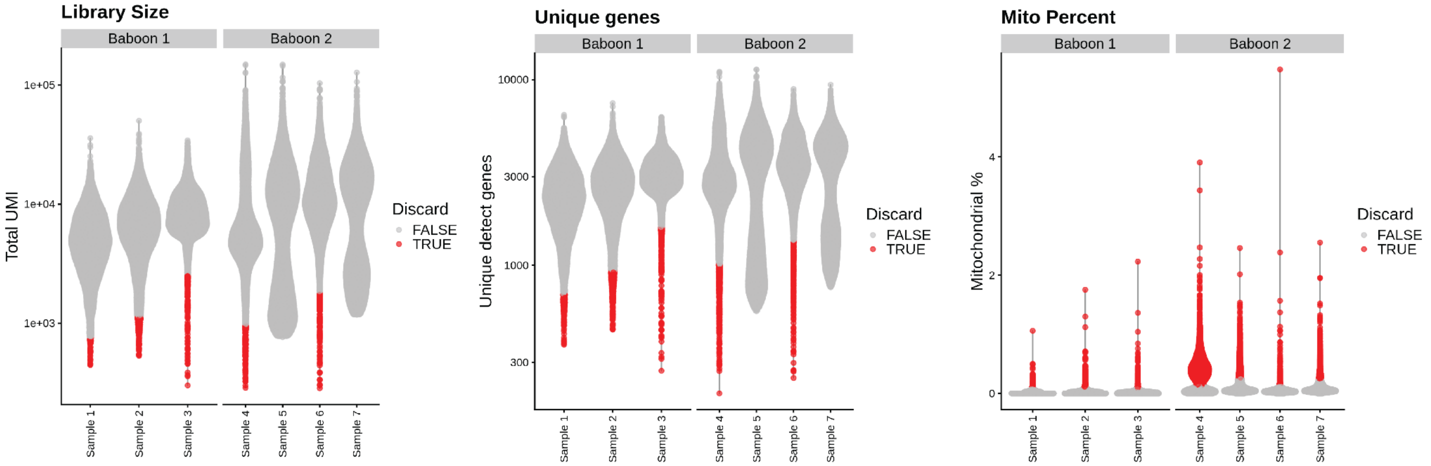

Supplementary Figure 3: Quality control metrics for baboon samples. Violin plots displaying library size, number of unique genes, and mitochondrial percent metrics of the seven baboon samples (n=7) across two unique donors. Red data points were considered low-quality nuclei and were excluded from downstream analyses.

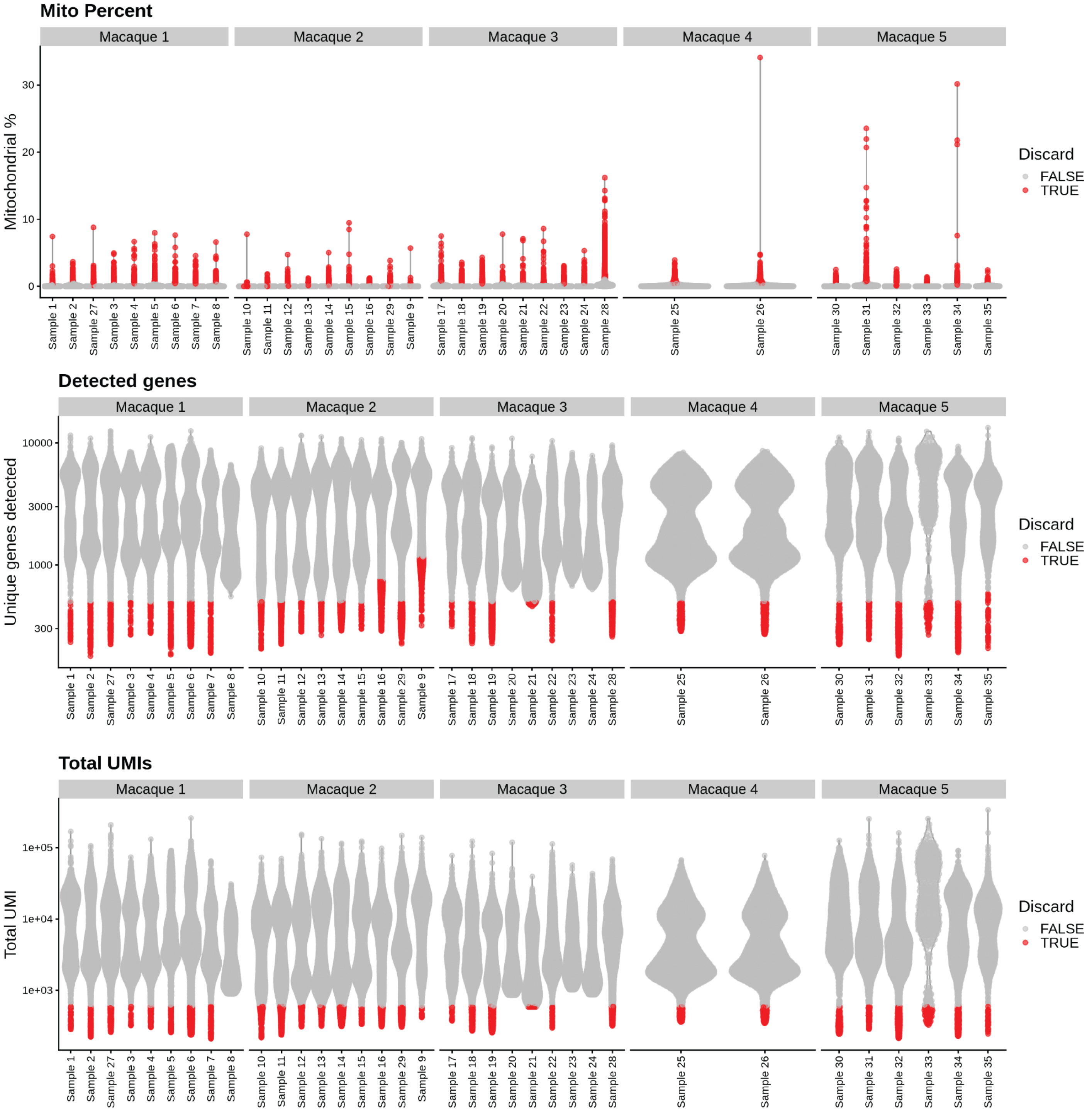

Supplementary Figure 4: Quality control metrics for macaque samples. Violin plots displaying library size, number of unique genes, and mitochondrial percent metrics of the thirty-five macaque samples (n=35) across five unique donors. Red data points were considered low-quality nuclei and were excluded from downstream analyses.

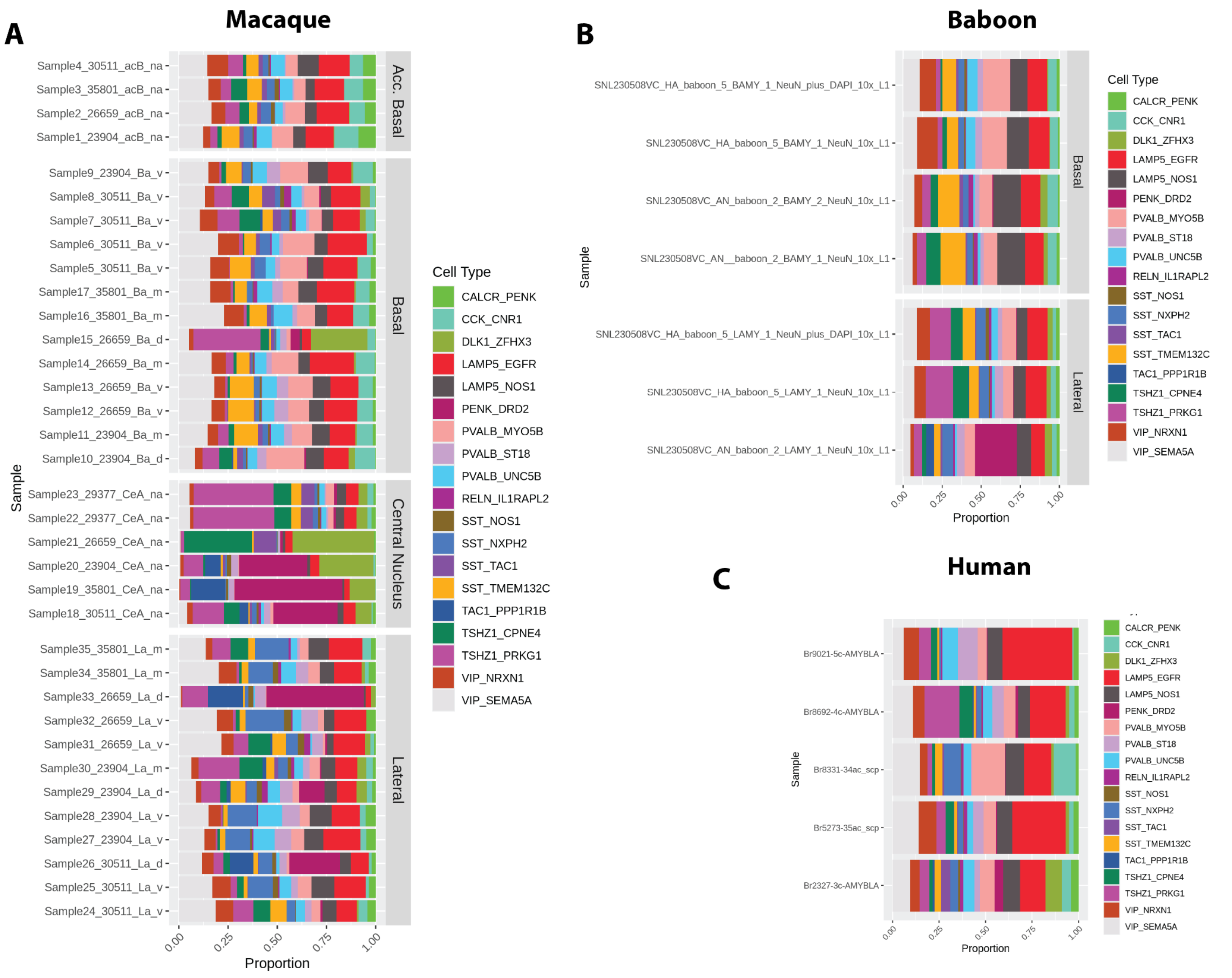

Supplementary Figure 5: Inhibitory neuron cell type proportions across all samples. Proportional distribution of fine inhibitory cell types across amygdala subdivisions in macaque, baboon, and human samples. (A) Stacked bar plots depict the proportion of each cell type within samples from different subdivisions of the macaque amygdala, including Accessory Basal, Basal, Central Nucleus, and Lateral punches. Sample naming scheme reflects the sample number, ONPRC ID#, subdivision sampled, and if the punch was dorsal (d), intermediate (m), or ventral (v). (B) Similar analysis for baboon samples, showing cell type distribution across Basal and Lateral punches. (C) Cell type proportions in human amygdala samples which sampled the whole basolateral amygdala region. Colors represent fine cell types, as indicated in the legend, highlighting conserved patterns of cell type distribution across subdivisions.

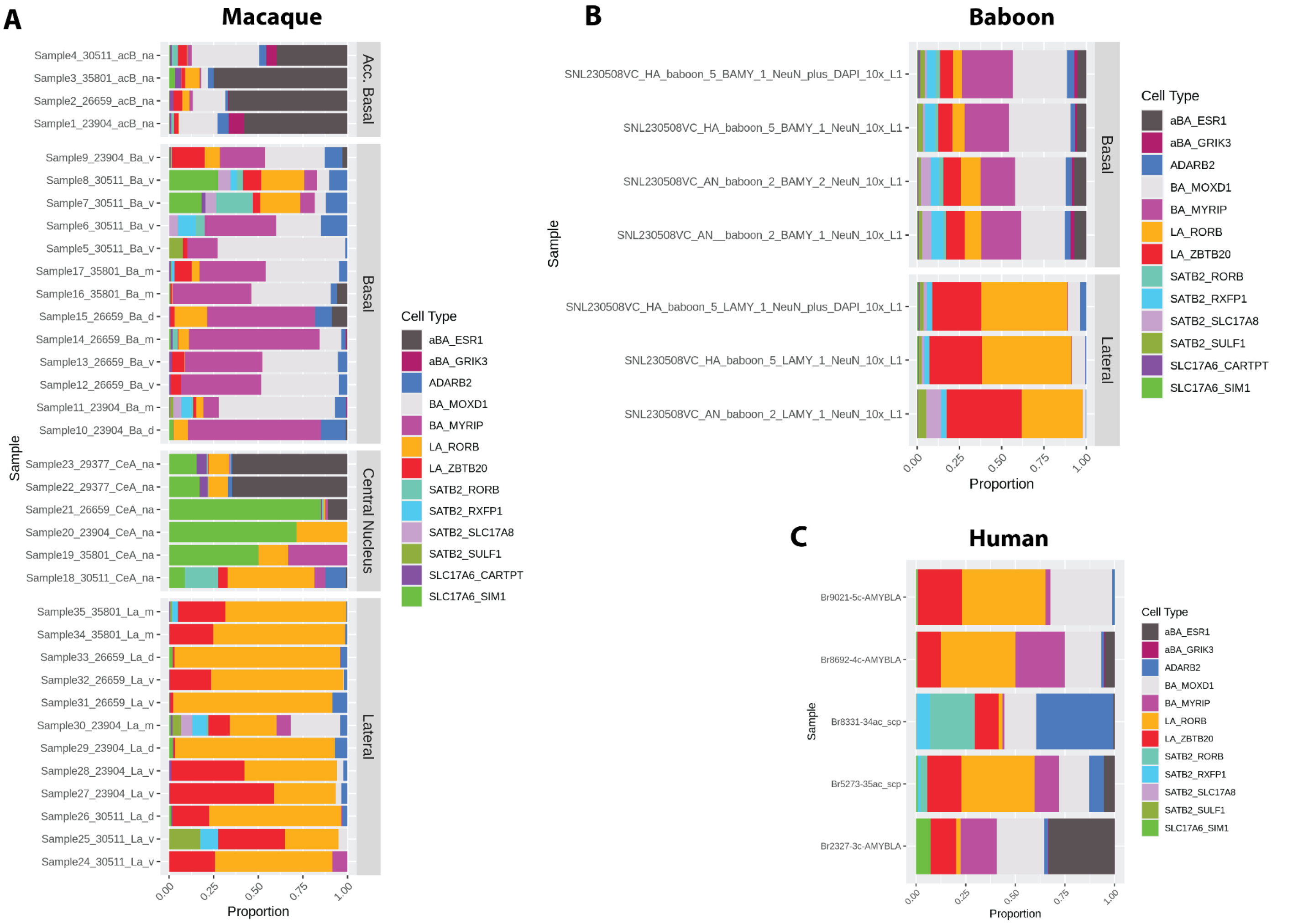

Supplementary Figure 6: Excitatory neuron cell type proportions across all samples. Proportional distribution of fine excitatory cell types across amygdala subdivisions in macaque, baboon, and human samples. (A) Stacked bar plots depict the proportion of each cell type within samples from different subdivisions of the macaque amygdala, including Accessory Basal, Basal, Central Nucleus, and Lateral punches. Sample naming scheme reflects the sample number, ONPRC ID#, subdivision sampled, and if the punch was dorsal (d), intermediate (m), or ventral (v). (B) Similar analysis for baboon samples, showing cell type distribution across Basal and Lateral punches. (C) Cell type proportions in human amygdala samples which sampled the whole BLA. Colors represent fine cell types, as indicated in the legend, highlighting conserved patterns of cell type distribution across subdivisions.

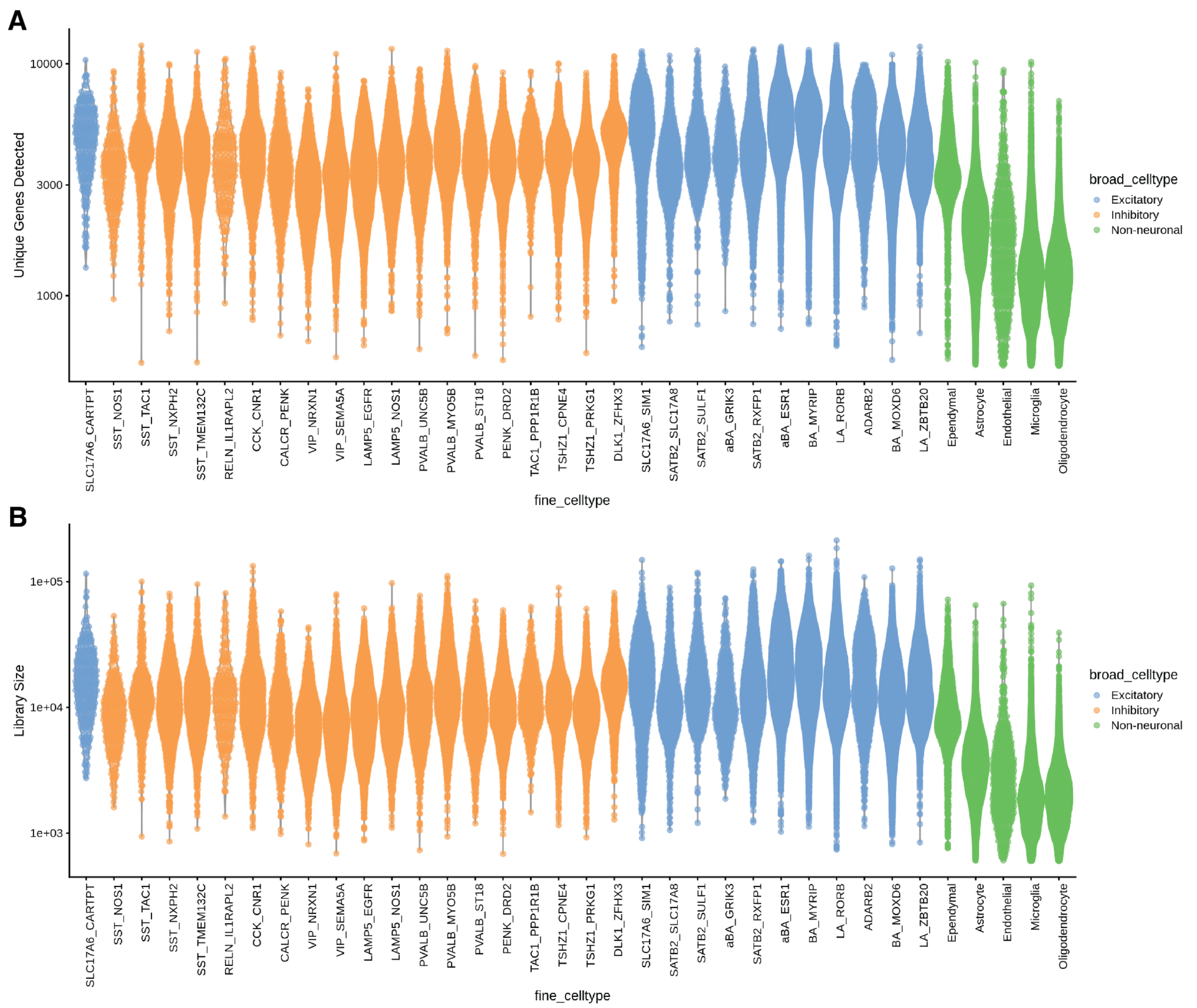

Supplementary Figure 7: Number of unique genes detected and library size across fine cell type clusters. Violin plots displaying the number of unique genes (A) and library size (B) across fine cell type clusters. Data points are colored by broad cell type clusters.

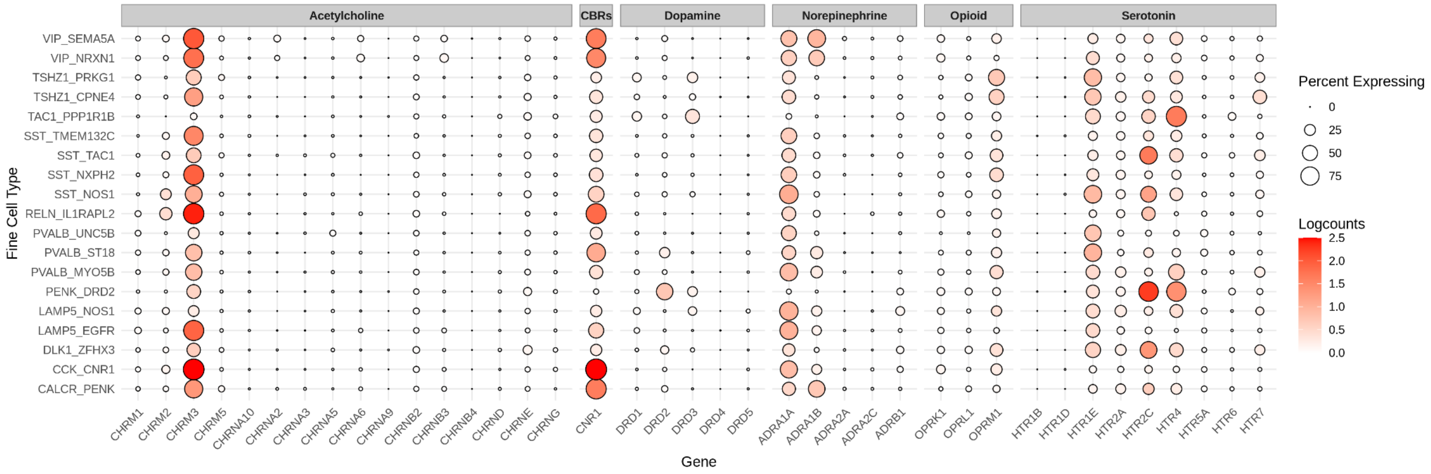

Supplementary Figure 8: Expression of neuromodulatory receptor genes across transcriptionally distinct inhibitory neuron types in the primate amygdala. Dot plot showing the average expression (color) and percent of cells expressing (dot size) key receptor genes for acetylcholine, GABA, dopamine, norepinephrine, opioid, and serotonin across 19 inhibitory neuron types. Distinct neuromodulatory receptor profiles were observed across cell types, highlighting the molecular diversity and potential differential modulation of inhibitory circuits in the amygdala.

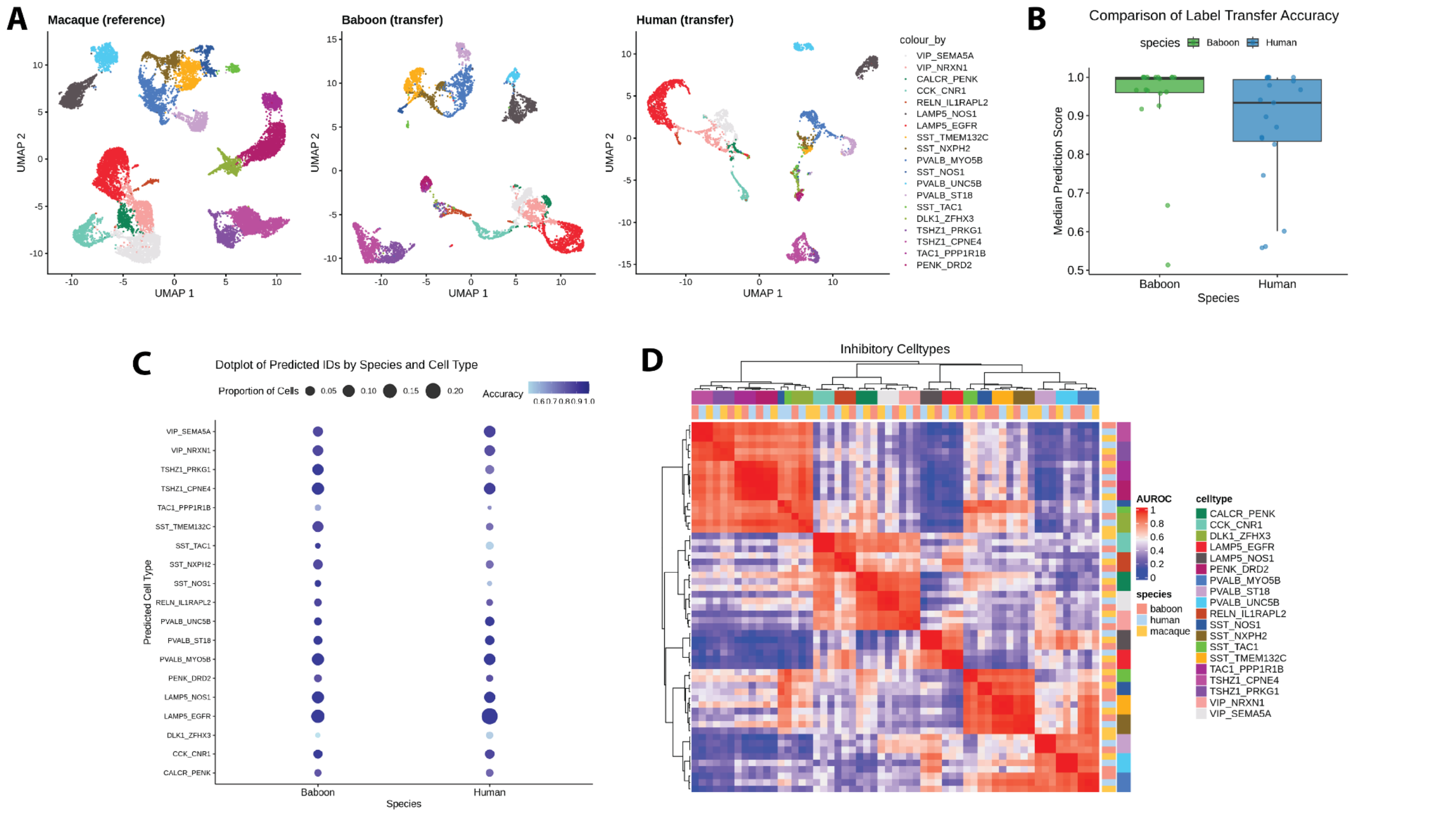

Supplementary Figure 9: Within-species clustering and label transfer reveals that amygdala inhibitory neurons are highly conserved across species. (A) UMAP of macaque inhibitory clusters (left) and label transfer results in human (center) and baboon (right), colored by predicted cell type. Cell type labels were defined in macaques and transferred to the other species using Seurat (see Methods). (B) Median prediction scores (label transfer accuracy) were high across cell types, indicating reliable cross-species classification. (C) Dot plot showing the proportion of cells assigned to each predicted cell type (dot size) and median prediction accuracy (color) by species. (D) Cross-species classification accuracy via MetaNeighbor revealed that amygdala inhibitory cell types are highly conserved across species.

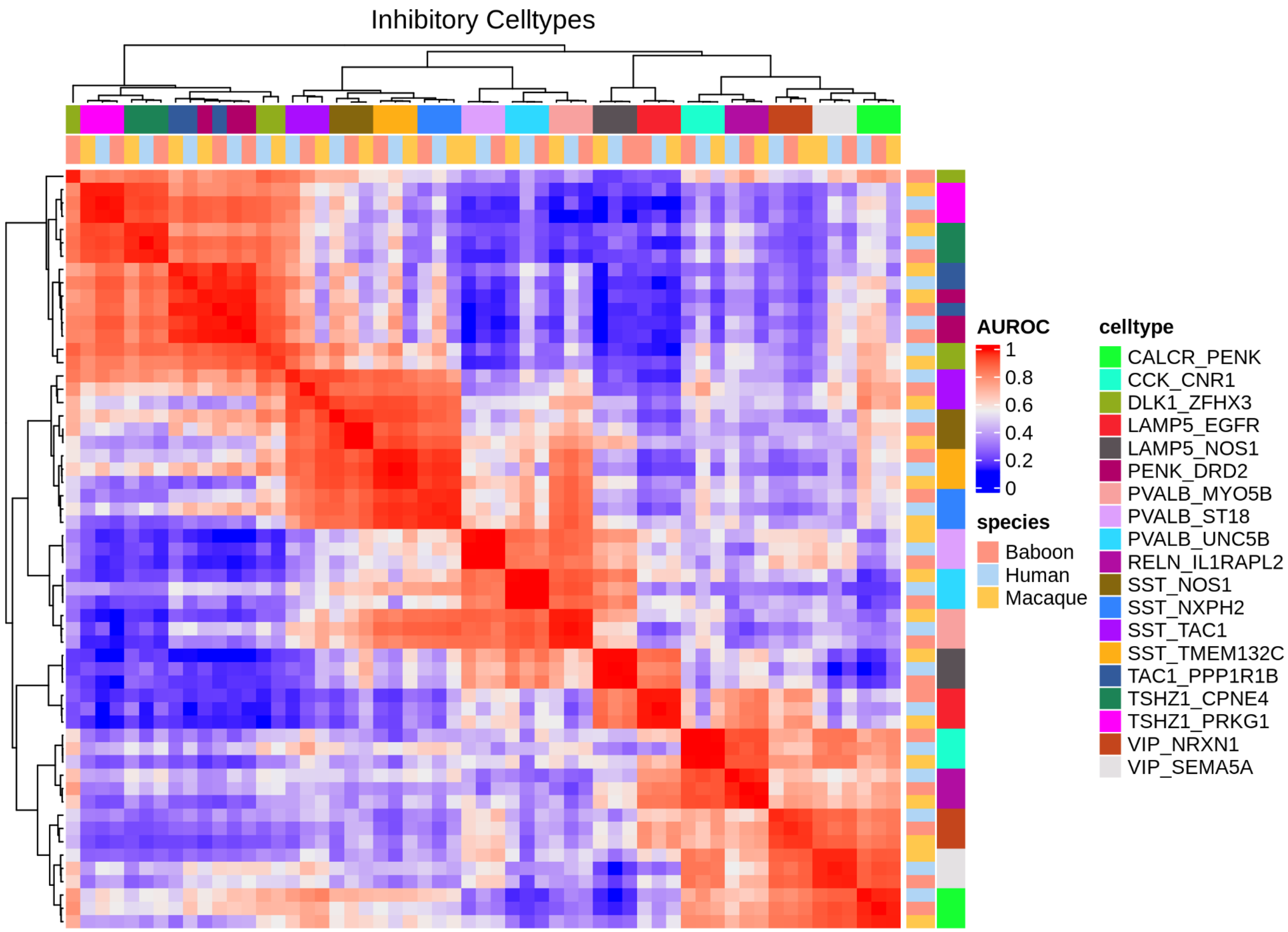

Supplementary Figure 10: Inhibitory neurons found in the amygdala across species. Heatmap displaying unsupervised MetaNeighbor cross-species cell type accuracy for inhibitory neurons. Inhibitory cell type classification accuracy was assessed across cell types across species. Color scale represents the area under the receiver operating characteristic (AUROC) curve where positive (red) values indicate higher-than-chance prediction accuracy. Rows and columns indicate target and test cell types, respectively.

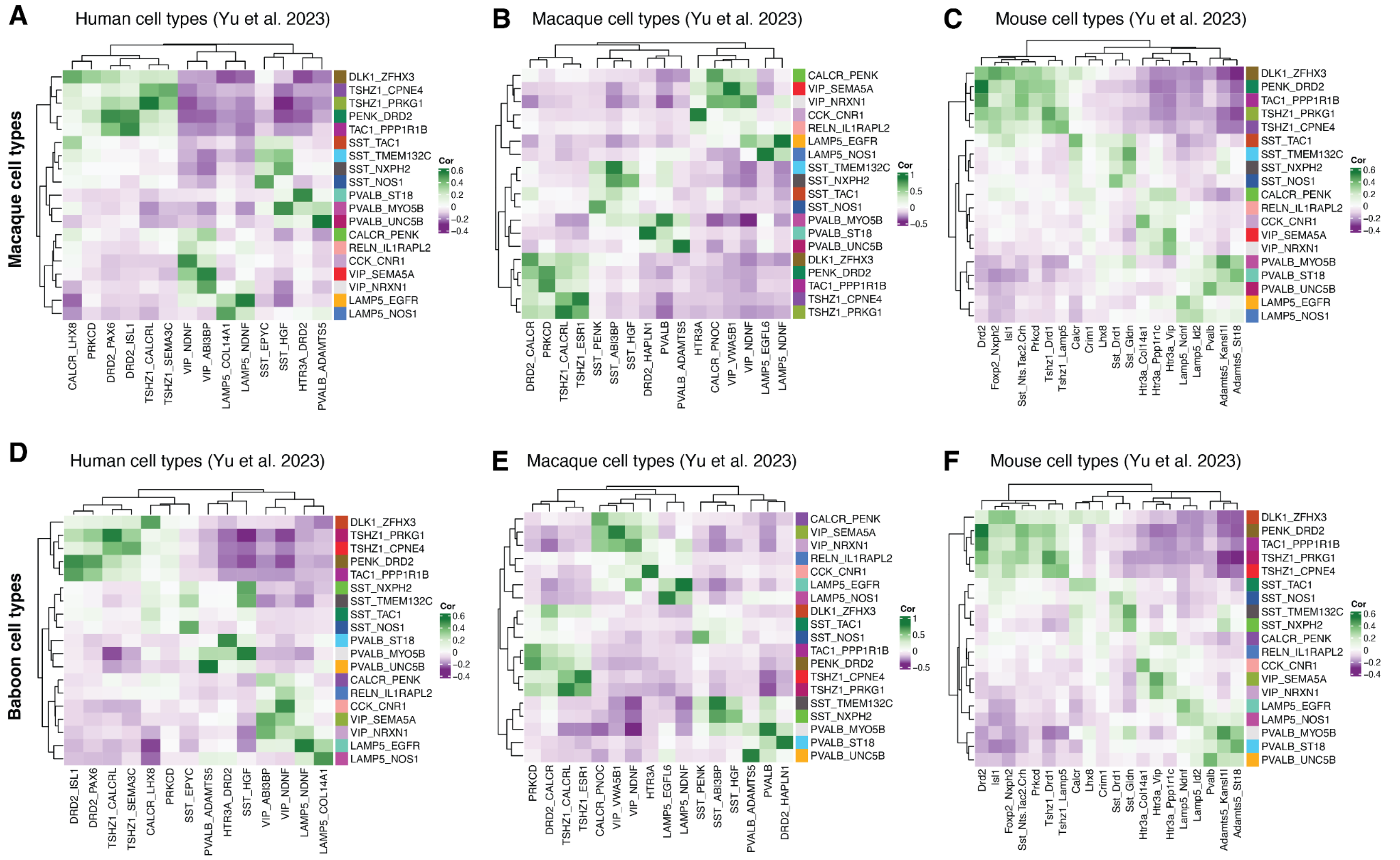

Supplementary Figure 11: Cross-species and dataset comparisons of inhibitory cell types found int he amygdala. Heatmaps show pairwise Pearson correlation coefficients between our inhibitory cell-type profiles (rows) and those from Yu et al. (*22*) reference datasets (columns). Panels A–C compare macaque inhibitory subtypes to human (A), macaque (B) and mouse (C), while panels D–F do the same for baboon inhibitory subtypes versus human (D), macaque (E) and mouse (F). Dendrograms on both axes reflect hierarchical clustering of cell type–specific expression patterns. Color intensity displays correlation strength (green = strong positive; white ≈ 0; purple = negative), highlighting conserved versus divergent transcriptional relationships among inhibitory neurons across species.

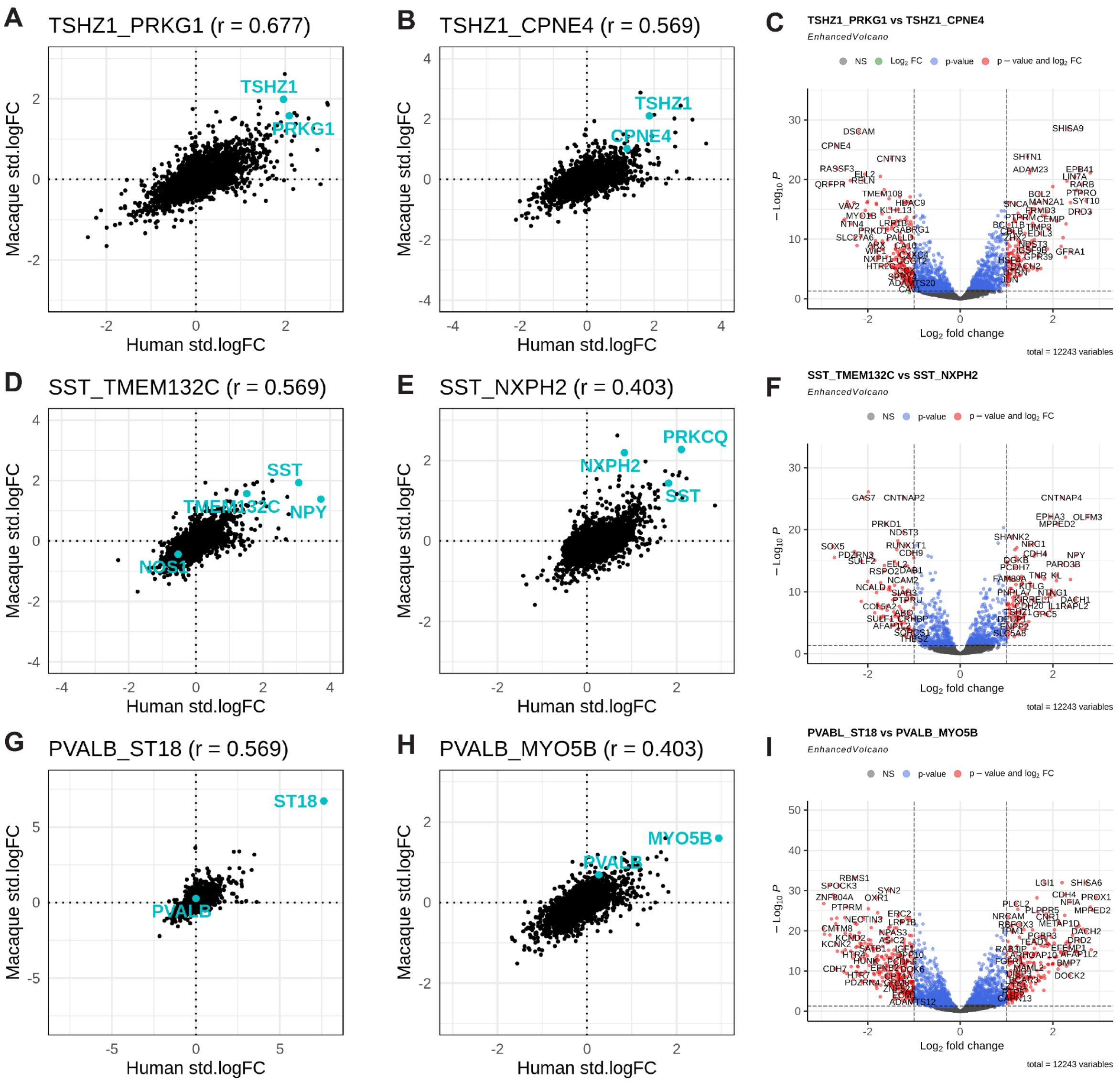

Supplementary Figure 12: Spatially and molecularly distinct inhibitory neuron types display conserved marker gene expression across primate species. (A–B) Correlation of standardized log fold-change (logFC) marker gene expression in human vs. macaque for two intercalated (*TSHZ1*+) cell types: *PRKG1*+ and *CPNE4*+. (C) Pseudobulk differential gene expression volcano plot comparing TSHZ1_PRKG1(positive logFC) vs. TSHZ1_CPNE4 (negative logFC). (D–E) Cross-species correlation of standardized logFC for two SST-expressing inhibitory neuron types: *TMEM132C*+ and *NXPH2*+. We note that *NPY* and *PRKCQ* are the top

conserved marker genes, respectively, for these populations. (F) Volcano plot comparing pseudobulked SST_TMEM132C (positive logFC) vs. SST_NXPH2 (negative logFC). (G–H) Cross-species correlation of standardized logFC for two PVALB-expressing neuron types: *ST18*$^{+}$ and *MYO5B*$^{+}$. (I) Volcano plot comparing pseudobulked PVALB_ST18 (positive logFC) vs. PVALB_MYO5B (negative logFC). Red data points in volcano plots represent genes with a |logFC| > 1 and FDR adjusted p-value < 0.05.

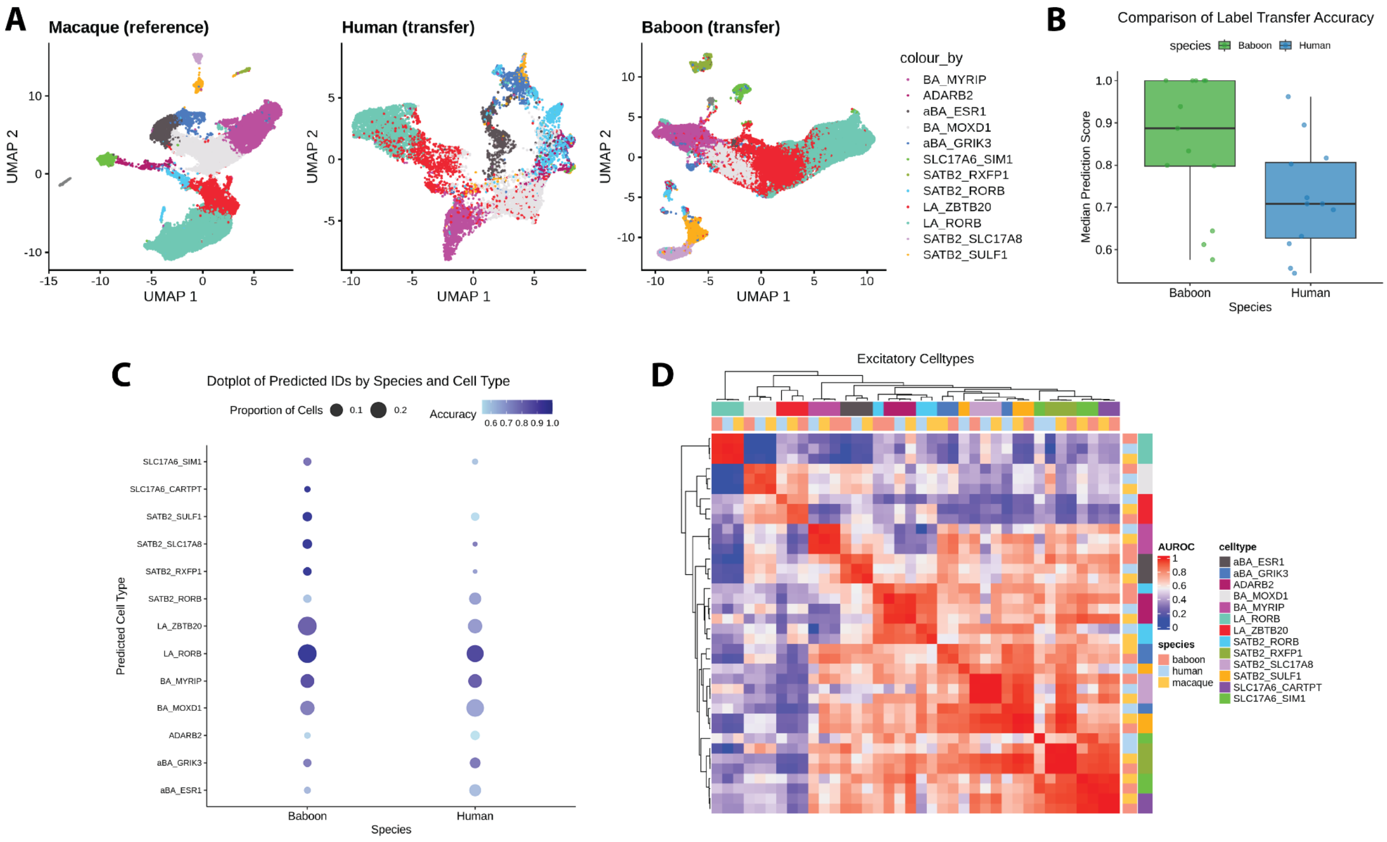

Supplementary Figure 13: Within-species clustering and label transfer reveals that amygdala excitatroy neurons are highly conserved across species. (A) UMAP of macaque excitatory clusters (left) and label transfer results in human (center) and baboon (right), colored by predicted cell type. Cell type labels were defined in macaques and transferred to the other species using Seurat (see Methods). (B) Median prediction scores (label transfer accuracy) were high across cell types, indicating reliable cross-species classification. (C) Dot plot showing the proportion of cells assigned to each predicted cell type (dot size) and median prediction accuracy (color) by species. (D) Cross-species classification accuracy via MetaNeighbor revealed that amygdala excitatory cell types are highly conserved across species.

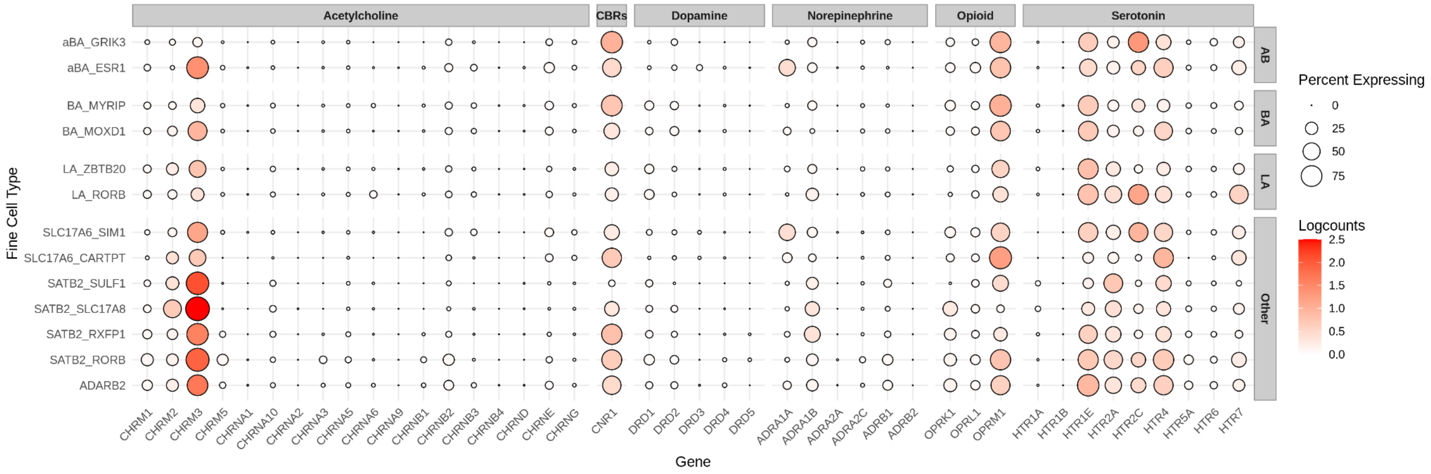

Supplementary Figure 14: Expression of neuromodulatory receptor genes across transcriptionally distinct excitatory neuron types in the primate amygdala. Dot plot showing the average expression (color) and percent of cells expressing (dot size) key receptor genes for acetylcholine, GABA, dopamine, norepinephrine, opioid, and serotonin across 13 excitatory neuron types, grouped by spatial localization. Distinct neuromodulatory receptor profiles were observed across cell types, highlighting the molecular diversity and potential differential modulation of excitatory circuits in the amygdala. aBA= accessory basal, BA = basal, LA = lateral.

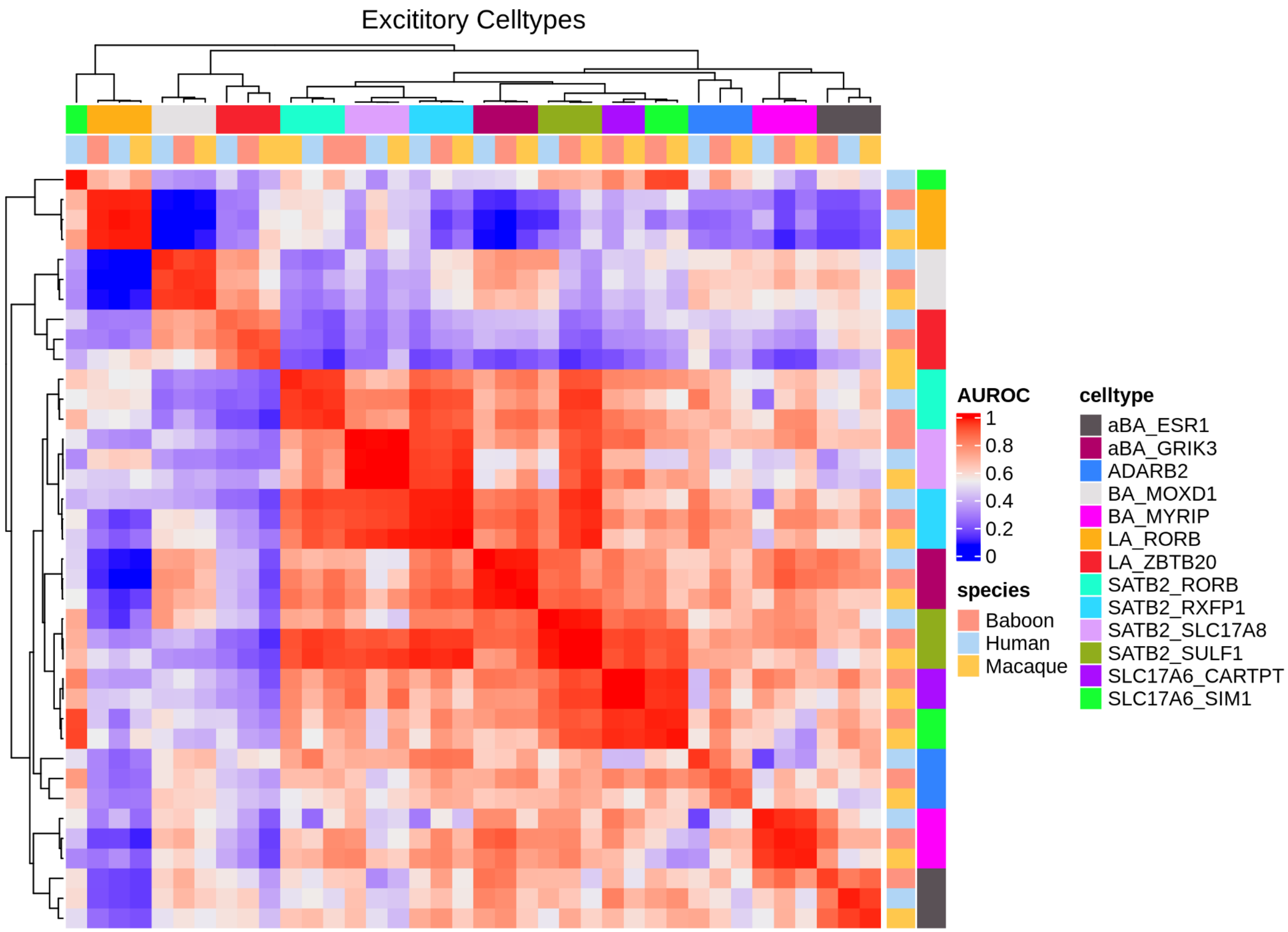

Supplementary Figure 15: Cross-species analysis of excitatory neurons using MetaNeighbor. (A) Heatmap displaying unsupervised MetaNeighbor cross-species cell type accuracy for excitatory neurons. Excitatory cell type classification accuracy was assessed across cell types across species. Color scale represents the area under the receiver operating characteristic (AUROC) curve where positive (red) values indicate higher-than-chance prediction accuracy. Rows and columns indicate target and test cell types, respectively.

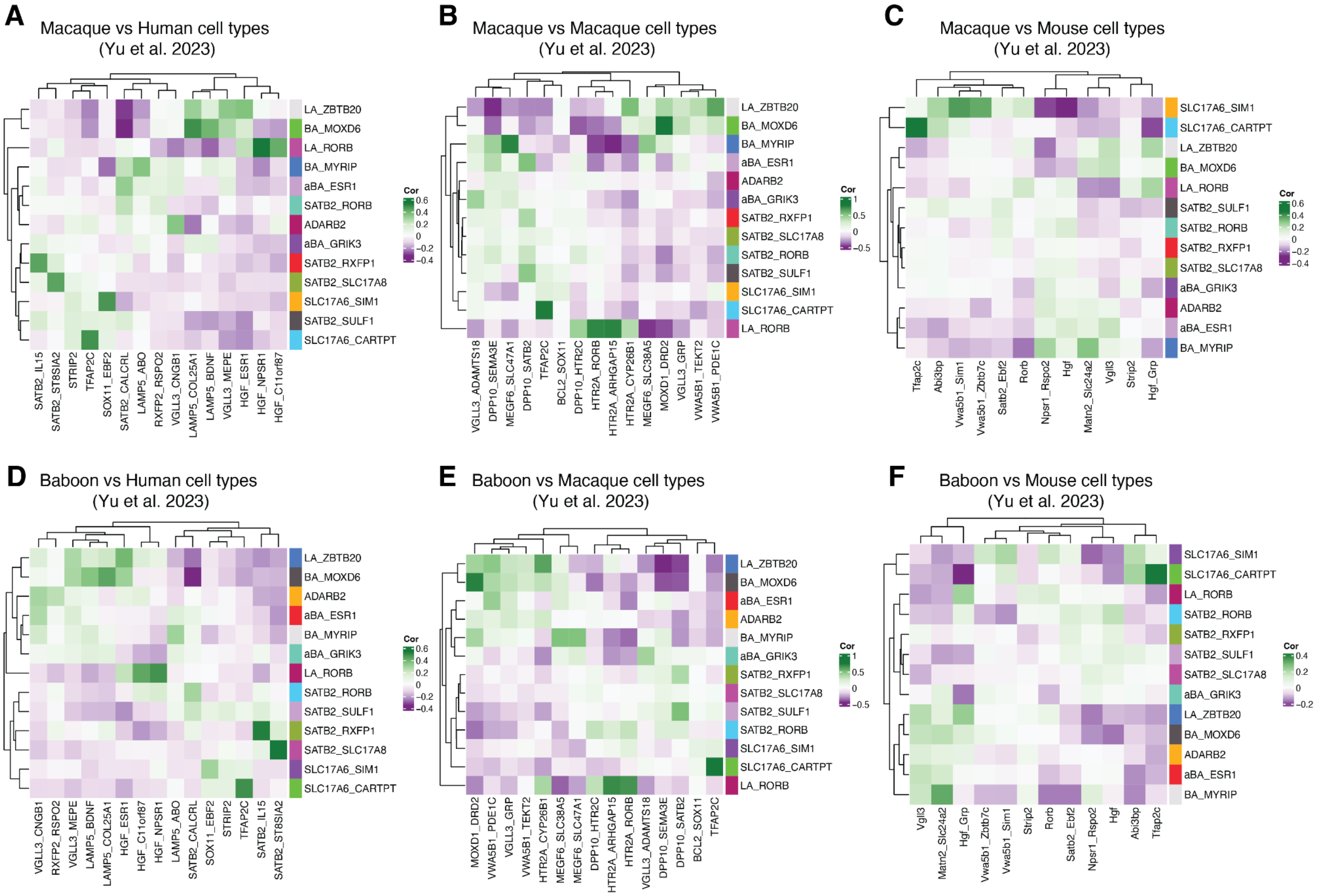

Supplementary Figure 16: Cross-species and dataset comparisons of excitatory cell types found int he amygdala. Heatmaps show pairwise Pearson correlation coefficients between our excitatory cell-type profiles (rows) and those from Yu et al. (*22*) reference datasets (columns). Panels A–C compare macaque excitatory subtypes to human (A), macaque (B) and mouse (C), while panels D–F do the same for baboon excitatory subtypes versus human (D), macaque (E) and mouse (F). Dendrograms on both axes reflect hierarchical clustering of cell type–specific expression patterns. Color intensity displays correlation strength (green = strong positive; white ≈ 0; purple = negative), highlighting conserved versus divergent transcriptional relationships among excitatory neurons across species.

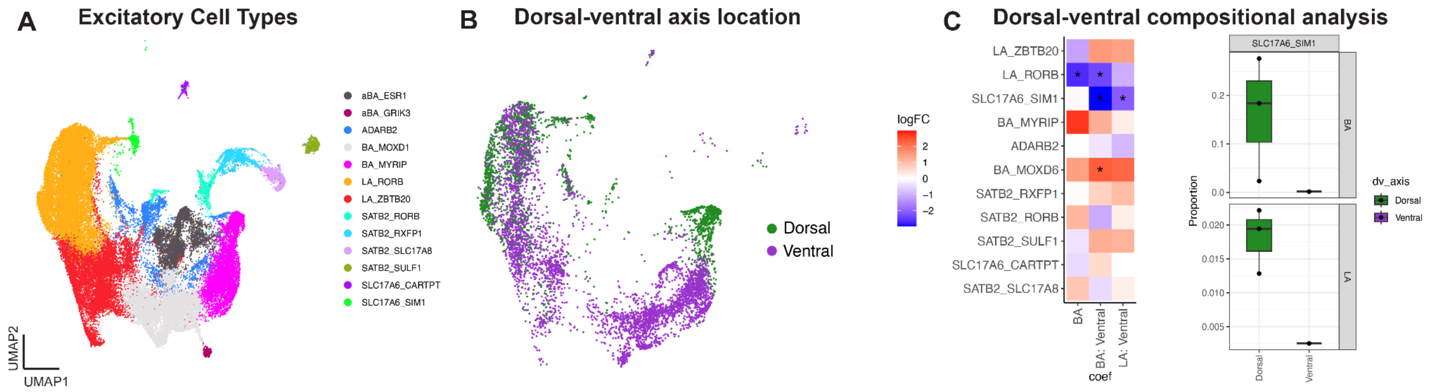

Supplementary Figure 17: Excitatory neurons expressing *SLC17A6* and *SIM1* were sampled from dorsal punches in NHPs. UMAP visualization colored by fine excitatory cell type cluster (A) and dorsal vs ventral punch locations (B) in macaques and baboons. Compositional analysis (C) shows that $SLC17A6^{+}/SIM1^{+}$ neurons were enriched in dorsal punches, suggesting that they are likely derived from partial sampling of the medial nucleus of the amygdala. '*' denotes FDR-adjusted p-value < 0.05.

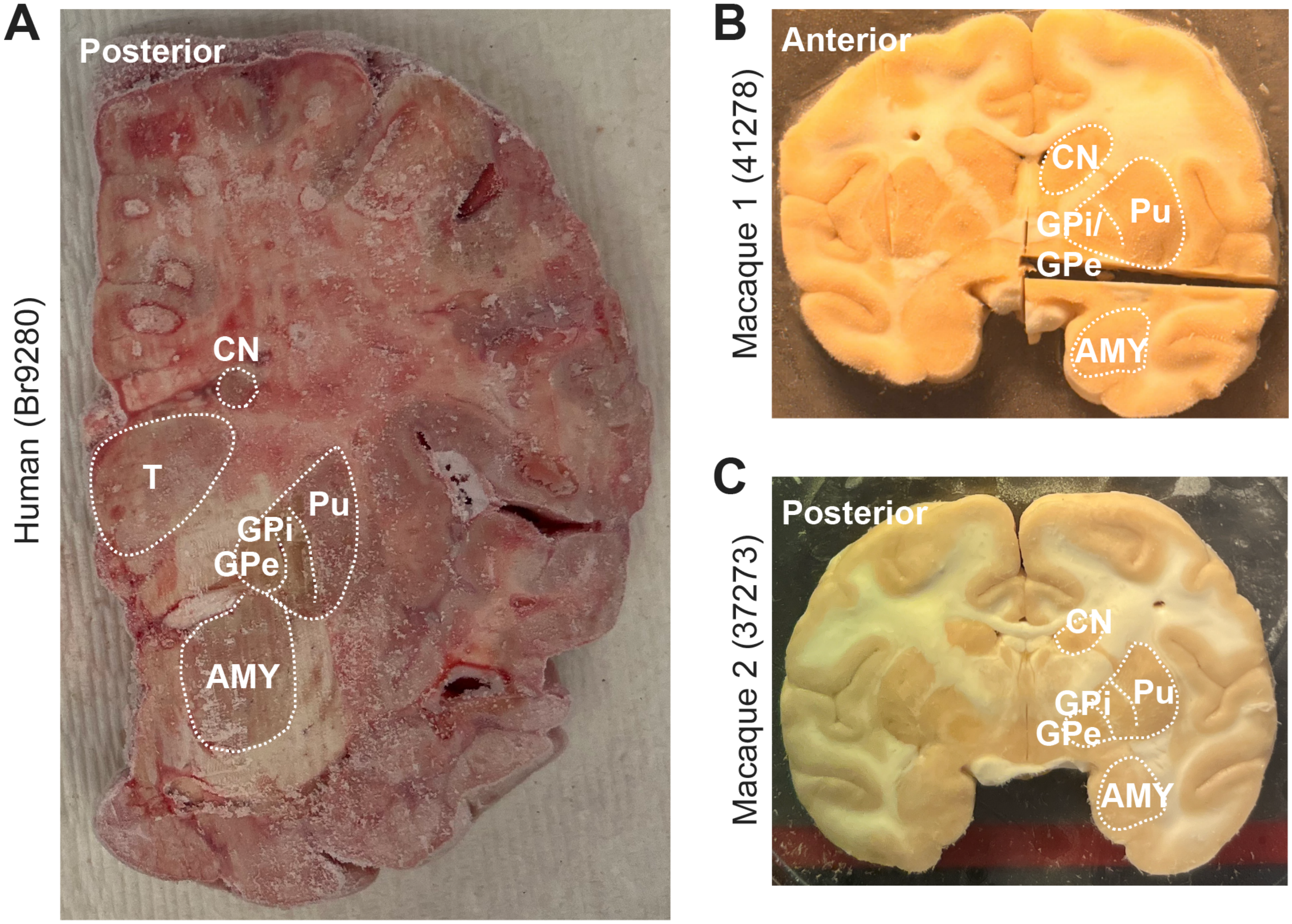

Supplementary Figure 18. Human and macaques fresh frozen coronal brain slabs used for smFISH validation. **(A)** Left human coronal brain hemisphere for donor Br9280 with indicated anatomical landmarks at the posterior level of the amygdala. **(B,C)** Coronal brain slabs for macaque 1 (41278) and macaque 2 (37273) with indicated anatomical landmarks at the anterior and posterior levels of the amygdala, respectively. AMY – amygdala, CN – caudate nucleus, GPe – globus pallidus external, GPi – globus pallidus internal, Pu – putamen, T – thalamus.

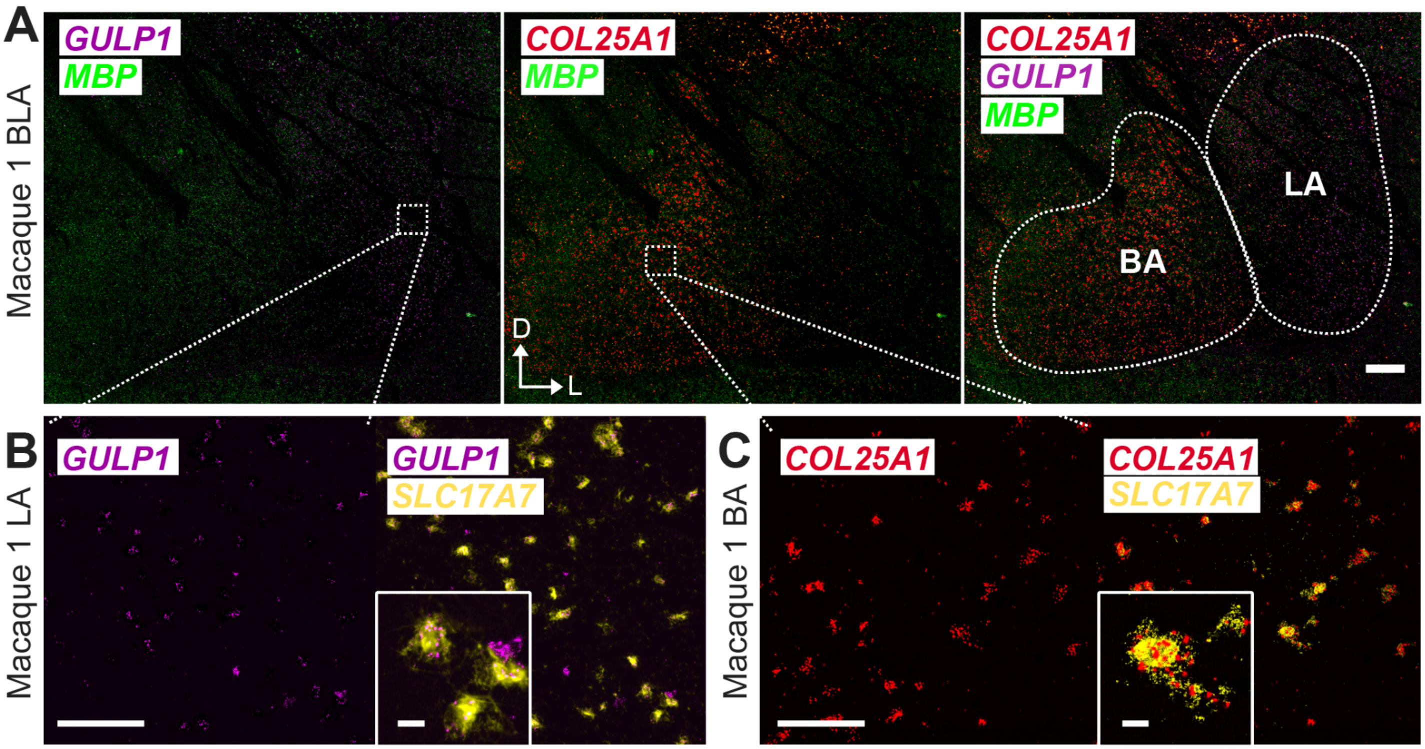

Supplementary Figure 19. *GULP1* marker gene expression for lateral amygdala (LA) and *COL25A1* marker gene expression for basal amygdala (BA) in macaque 1. (A) 2X smFISH images of the BLA region of the macaque brain illustrating expression of *GULP1* (magenta) in LA and *COL25A1* (red) in BA. *MBP* (green) represents white matter for anatomical landmarks. Dorval (D) and lateral (L) arrows are added for tissue directionality. White boxes represent approximate locations of zoomed-in images. Scale bar 1000 µm. **(B)** Zoomed in 40X smFISH images illustrating co-expression of *GULP1* (magenta) and *SLC17A7* (yellow) within the LA. Scale bar 100 µm. Inset illustrates representative neurons co-expressing *GULP1* and *SLC17A7*. Scale bar of inset 10 µm. **(C)** Zoomed in 40X smFISH images illustrating co-expression of *COL25A1* (red) and *SLC17A7* (yellow) within the BA. Scale bar 100 µm. Inset illustrates a representative neuron co-expressing *COL25A1* and *SLC17A7*. Scale bar of inset 10 µm.

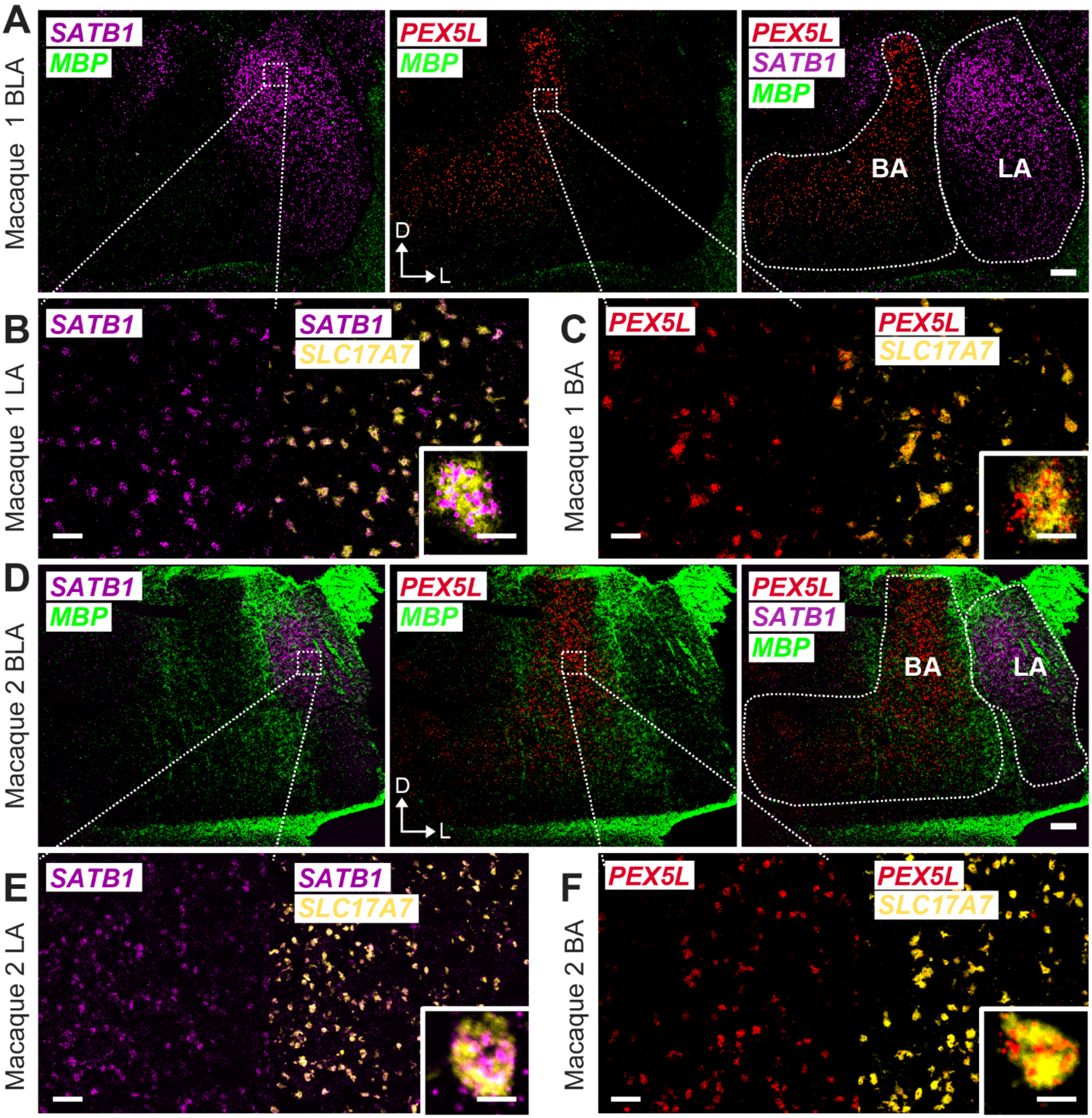

Supplementary Figure 20. ***SATB1*** **marker gene expression for lateral amygdala (LA) and** ***PEX5L*** **marker gene expression for basal amygdala (BA) in two macaque brains. (A, D)** 2X smFISH images of the BLA region of two macaque brains illustrating expression of *SATB1* (magenta) in LA and *PEX5L* (red) in BA. *MBP* (green) represents white matter for anatomical landmarks. Dorval (D) and lateral (L) arrows are added for tissue directionality. White boxes represent approximate locations of zoomed-in images. Scale bar 500 µm. **(B, E)** 40X smFISH images illustrating co-expression of *SATB1* (magenta) and *SLC17A7* (yellow) within the macaque LA. Scale bar 50 µm. Inset illustrates a representative neuron co-expressing *SATB1* and *SLC17A7*. Scale bar of inset 10 µm. **(C, F)** 40X smFISH images illustrating co-expression of *PEX5L* (red) and *SLC17A7* (yellow)

within the macaque BA. Scale bar 50 µm. Inset illustrates a representative neuron co-expressing *PEX5L* and *SLC17A7*. Scale bar of inset 10 µm.

ONPRC ID	Species	Age	Sex	Assay
29377	Macaca mulatta	10.78	F	snRNA-seq
30511	Macaca mulatta	10.51	F	snRNA-seq
26659	Macaca mulatta	14.23	F	snRNA-seq
23904	Macaca mulatta	18.2	M	snRNA-seq
35801	Macaca mulatta	5.56	M	snRNA-seq
32273	Macaca mulatta	5.97	F	RNAscope
41278	Macaca mulatta	2.12	M	RNAscope
39944	Papio anubis	3.62	F	snRNA-seq
39947	Papio anubis	4.32	F	snRNA-seq

Supplementary Table 1. Demographic information on the nonhuman primate tissue samples. Demographic information including animal number ID in the ONPRC medical records database, species, age at time of death, sex, and assay for which the brain was utilized.

Brain ID	Sex	Race	Age	Best RIN PFC	PMI	Assay
Br5273	F	Caucasian	53.89	7.0	34.5	snRNA-seq
Br8331	M	Hispanic	47.5	7.2	12.5	snRNA-seq
Br2723	M	Caucasian	50.4	7.1	25.5	snRNA-seq
Br9021	M	African American	70.74	7.6	15.5	snRNA-seq
Br8692	M	African American	48.21	8.3	10.5	snRNA-seq
Br9280	M	Caucasian	66.6	9.3	25.5	RNAscope

Supplementary Table 2. Human brain donor demographic information. Demographic information on brain donors including Brain ID, sex (F, female; M, male), race (CAUC, Caucasian; HISP, Hispanic; AA, African American), age at the time of death, best RNA integrity number (RIN) measured in the prefrontal cortex (PFC), post-mortem interval (PMI), and assay for which the brain was utilized.

Abbreviation	Definition
aBA	Accessory basal nucleus
BA	Basal nucleus
Bi	Intermediate region of the basal nucleus
BLA	Basolateral amygdaloid complex
Bmc	Magnocellular region of the basal nucleus
Bpc	Parvocellular region of the basal nucleus
BNST	Bed nucleus of the stria terminalis
CeA	Central nucleus
CN	Caudate nucleus
GPe	Globus pallidus external
GPi	Globus pallidus internal
ITC	Intercalated cell masses
LA	Lateral nucleus
Ld	Dorsal region of the lateral nucleus
LGE	Lateral ganglionic eminence
Lv	Ventral region of the lateral nucleus
MeA	Medial amygdala
Pu	Putamen

Supplementary Table 3. Anatomical abbreviations.
